# Extracellular Mitochondrial‐Derived Vesicles Affect the Progression of Diabetic Foot Ulcer by Regulating Oxidative Stress and Mitochondrial Dysfunction

**DOI:** 10.1002/advs.202407574

**Published:** 2025-01-21

**Authors:** Huihui Zhang, Zi Yan, Junyou Zhu, Ziyue Li, Lianglong Chen, Weihan Zheng, Zhenning Dai, Jiaxin Yang, Xinyi Yun, Yilin Wang, Hai Zhou, Ziwei Jiang, Qiuyi Yu, Shiyu Li, Wenhua Huang, Lei Yang

**Affiliations:** ^1^ Department of Burns Nanfang Hospital Southern Medical University Guangzhou 510515 China; ^2^ Guangdong Engineering Research Center for Translation of Medical 3D Printing Application Guangdong Provincial Key Laboratory of Digital Medicine and Biomechanics National Key Discipline of Human Anatomy School of Basic Medical Sciences Southern Medical University Guangzhou 510515 China; ^3^ Department of Microbiology and Immunology College of Basic Medicine and Public Hygiene Jinan University Guangzhou 510632 China; ^4^ Guangdong Medical Innovation Platform for Translation of 3D Printing Application The Third Affiliated Hospital of Southern Medical University Southern Medical University Guangzhou 510630 China; ^5^ Department of Burns First affiliated hospital Sun Yat‐sen University Guangzhou 510080 China; ^6^ Department of Stomatology Guangdong Provincial Key Laboratory of Research and Development in Traditional Chinese Medicine Guangdong Second Traditional Chinese Medicine Hospital Guangzhou 510095 China

**Keywords:** diabetic foot ulcers, extracellular vesicles, mitochondria, mitochondrial‐derived vesicles, oxidative stress

## Abstract

Diabetic foot ulcer (DFU) is a common and severe complication of diabetes mellitus, the etiology of which remains insufficiently understood, particularly regarding the involvement of extracellular vesicles (EVs). In this study, nanoflow cytometry to detect EVs in DFU skin tissues is used and found a significant increase in the Translocase of Outer Mitochondrial Membrane 20 (TOM20)^+^ mitochondrial‐derived vesicles (MDVs). The role of MDVs in DFU is yet to be reported. Using single‐cell datasets, it is discovered that the increase in MDVs may be regulated by Sorting Nexin 9 (SNX9). In vitro experiments revealed that MDVs secreted by fibroblasts cultured in high glucose medium exhibited similar composition and protein enrichment results to those in DFU tissues, suggesting their potential as an ideal in vitro surrogate. These MDVs promoted apoptosis and intracellular oxidative stress, disrupted mitochondrial structure, and reduced aerobic metabolism in target cells. In vivo experiments also showed that MDV drops hindered wound healing in diabetic mice; however, this effect is rescued by SNX9 inhibitors, restoring mitochondrial dynamics and balance. Under high glucose conditions, MDVs significantly upregulated oxidative stress levels and induced mitochondrial dysfunction. This study proposes targeting MDVs as a potential therapeutic strategy for DFU.

## Graphical abstract

Extracellular vesicles (EVs) play a crucial role in diabetic foot ulcer (DFU) pathology. This study identified increased TOM20^+^ mitochondrial‐derived vesicles (MDVs) in DFU tissues, regulated by Sorting Nexin 9 (SNX9). In vitro and in vivo experiments showed these MDVs promote oxidative stress, mitochondrial dysfunction, and hinder wound healing. SNX9 inhibitors restored balance, suggesting MDVs as targets for DFU treatment.

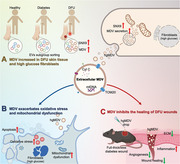

## 1 Introduction

Diabetic wounds, especially diabetic foot ulcers (DFU), are common complications of diabetes that significantly impact patients' quality of life. Patients with diabetes have a 30% lifetime risk of developing foot ulcers, and ≈85% of all lower‐limb amputations are preceded by foot ulcers.^[^ ^1^ ^]^ Despite recent advancements in treating DFU and an improved understanding of its healing mechanisms, the underlying molecular process remains unclear.^[^ ^2^ ^]^ Extracellular vesicles (EVs) are vital secretory products that mediate cell‐to‐cell communication and influence DFU.^[^ ^3^ ^]^ Bidirectional cell‐to‐cell communication mediated by EV‐borne cargo has emerged as a critical mechanism for wound healing.^[^ ^4^ ^]^ However, EVs demonstrate intricate heterogeneity and mediate various functions across physiological and pathological processes.^[^ ^5^, ^6^ ^]^ Given this complexity, the specific types of EVs significantly contributing to DFU healing remain unclear.

Mitochondria are critical organelles that maintain cellular metabolism and function. Their dynamics have been strongly linked to vascular damage and the hyperglycemia‐induced formation of reactive oxygen species (ROS) in DFU.^[^ ^7^, ^8^ ^]^ Moreover, the mitochondria serve as a source of EVs. Mitochondria‐derived vesicles (MDVs), generated through mitochondrial membrane budding, are a type of these vesicles.^[^ ^9^ ^]^ MDVs are ≈60–150 nm in size and generated in the inner or outer mitochondrial membranes.^[^ ^10^ ^]^ Notably, electron microscopy analysis revealed two distinct types of MDVs: single‐membraned MDVs, exclusively formed by the outer mitochondrial membrane, and double‐membraned MDVs with contents from the outer and inner mitochondrial membranes.^[^ ^11^ ^]^ These MDVs preferentially contain compartment‐specific proteins.^[^ ^12^ ^]^ As a mechanism for mitochondrial quality control, MDVs were initially identified as a potential method of eliminating damaged mitochondrial components.^[^ ^13^, ^14^ ^]^ MDV generation occurs at basal levels under normal physiological conditions^[^ ^15^, ^16^ ^]^ and is augmented in response to pathological stress. Stress conditions, such as remote ischemic preconditioning,^[^ ^17^ ^]^ oxidative stress,^[^ ^18^ ^]^ hypoxia,^[^ ^19^ ^]^ cannabidiol treatment,^[^ ^20^ ^]^ lipopolysaccharide,^[^ ^21^ ^]^ and heat stress,^[^ ^21^ ^]^ promote the selective incorporation of mitochondrial contents into MDVs. An increase in ROS production damages nucleic acids, proteins, and lipids. Consequently, cells may initiate the formation of MDV to facilitate the clearance of damaged mitochondrial particles.^[^ ^16^, ^22^ ^]^ A gradual accumulation of mitochondrial damage is the primary driver of MDV formation; however, the effects of these factors on the release of MDVs into the extracellular space warrant further investigation.

The fate of MDVs (peroxisomal‐ or lysosome‐mediated degradation or release into the extracellular space) is regulated by a complex mechanism.^[^ ^12^ ^]^ The release of MDVs into the extracellular space is primarily facilitated by two pathways: the multivesicular body‐mediated^[^ ^10^, ^14^, ^23^ ^]^ and the microvesicle pathway.^[^ ^21^, ^24^ ^]^ Sorting Nexin 9 (SNX9) is crucial in MDV formation and its subsequent delivery into lysosomes. The shRNA‐mediated knockdown of SNX9 significantly diminished the production of MDVs induced by mitochondrial damage, particularly in autophagy‐deficient cells.^[^ ^21^ ^]^ Reportedly, MDVs enriched with oxidized hSOD1 may bypass lysosomal degradation and be released into the extracellular space via EVs, thereby promoting aging.^[^ ^25^ ^]^ Furthermore, MDVs are encapsulated within multivesicular bodies and released into the extracellular space to protect cardiomyocytes against severe damage^[^ ^18^ ^]^ and facilitate intercellular communication with beneficial or harmful effects on recipient cells, based on the cellular source, the nature of the cargo, and the originating stimulus.^[^ ^26^ ^]^ The release and expulsion of MDV cargoes from cells allow them to act as signals capable of eliciting responses across different cell types and tissues.^[^ ^27^ ^]^ These MDVs significantly promote cell‐to‐cell communication^[^ ^28^, ^29^ ^]^ and may potentially affect the pathological processes associated with DFU. However, the MDV secretion mechanisms remain poorly understood.^[^ ^30^ ^]^ Although MDV formation has been confirmed to participate in oxidative stress^[^ ^31^ ^]^ and immune responses,^[^ ^32^ ^]^ the role of MDVs in the DFU progression process remains obscure. Nonetheless, our current understanding of the role of MDVs in diabetes and DFU remains limited.

In the present study, we successfully extracted MDVs from skin tissues and cell culture supernatants. Proteomics was used to identify the proteins carried by MDVs in both healthy individuals and patients with DFU. Herein, we investigated the effect of DFU on the composition of MDVs and the impact of MDVs on the DFU healing process. Elevated levels of MDVs were observed in patients with DFU compared with those in healthy controls. By measuring the relevant indicators, we observed that MDVs reduced cellular metabolism, heightened oxidative stress, and slowed the healing of diabetic wounds. To the best of our knowledge, this study is the first to suggest the involvement of MDVs in the progression of DFU and provide novel insights into the mechanisms underlying DFU development. Using both in vivo and in vitro models, we identified new pathways implicated in DFU pathogenesis and established a theoretical framework for identifying novel therapeutic targets in DFU treatment.

## 2 Results

### 2.1 MDVs in the DFU Skin Tissue were Significantly Increased

**Figure 1:**
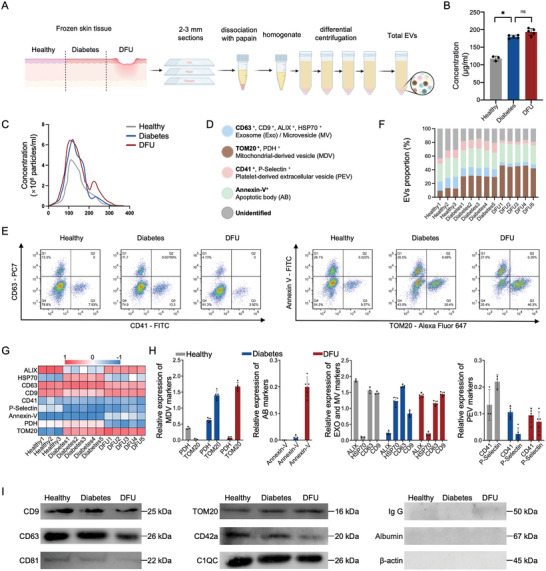
EV isolation and typing from Healthy, Diabetes, and DFU skin tissues. A) Schematic of EV isolation protocol from human skin tissue. B) Protein level of EVs was evaluated using a BCA protein assay kit (n = 3). C) Size distribution of EV particles. D) Schematic diagram of EXO/MV, MDV, PEV, and AB biomarkers. E) Bivariate dot‐plots of EVs using nanoflow cytometry and dual immunofluorescent staining with CD63/CD41 and TOM20/Annexin V. F) Quantitative analysis of nanoflow. G) Heat map of the protein microarray, displaying the expression level of EVs biomarkers in the samples. H) Quantitative analysis of EV biomarkers (n = 3). I) Western blot analysis of EVs extracted from skin tissue. Data are presented as the mean ± SD. Statistical analysis was performed using one‐way ANOVA. *: *p* < 0.05, ns: *p* > 0.05.

The experimental workflow is summarized in **Figure** **1**A. EV samples from healthy individuals, patients with diabetes, and DFU were isolated using differential centrifugation at ≈100 000 g. Protein concentration was measured using the BCA method to determine the number of EVs extracted. As shown in Figure 1B, the DFU (190.54 ± 8.04 µg mL^−1^) and Diabetes groups (177.12 ± 5.27 µg mL^−1^) have a higher EV content compared with the Healthy group (113.77 ± 6.41 µg mL^−1^). Nanoparticle tracking analysis (NTA) revealed that the size distribution of the EVs from each group ranged from 70–200 nm; small peaks in the 210–230 nm range may have been caused by non‐dispersed EVs (Figure 1C). The concentration determined by NTA was consistent with the results of the BCA method; there were more EVs in the skin tissues of the DFU and Diabetes groups. To clarify the EV subpopulations, we detected the expression of CD63, Translocase of Outer Mitochondrial Membrance 20 (TOM20), CD41, and annexin V as biomarkers of exosomes/microvesicles (EXO/MVs), MDVs, platelet‐derived EVs (PEVs), and apoptotic bodies (ABs), respectively (Figure 1D). We performed two dual immunofluorescence nanoflow cytometry analyses on the same EV samples, as shown in Figure 1E,F. The MDV subpopulations expressing TOM20 alone in Healthy, Diabetes, and DFU groups were 9.5 ± 0.7%, 30.4 ± 0.9%, and 46.3 ± 1.8%, respectively.

Marker protein levels in skin tissue EVs demonstrated a specific trend using protein microarray technology (Figure S1, Supporting Information). Differentially expressed markers are shown in a heat map (Figure 1G). The relative expression levels of each marker are shown in Figure 1H. TOM20 expression was higher in the DFU group than in the Diabetes and Healthy groups. However, Pyruvate Dehydrogenase (PDH), another MDV marker, was highly expressed in the Diabetes and Healthy groups, suggesting the existence of different MDV subtypes within each group. The expression levels of the biomarkers in the protein microarrays were consistent with the flow cytometry results. EXOs/MVs were expressed in all groups, whereas expression levels of ABs and PEVs were relatively low. Furthermore, we also detected EV markers using western blotting. CD9, CD63, CD81, and CD41a expression progressively decreased in the Healthy, Diabetes, and DFU groups, respectively. In contrast, the expression of TOM20 increased sequentially across the three groups. Complement C1q C chain (C1QC), an AB marker, showed consistent expression levels among the groups. This indicates that the western blotting results are consistent with those from the protein microarrays, confirming the presence of various subpopulations such as exosomes, microvesicles, platelet‐derived vesicles, and ABs in all groups. Additionally, the near absence of immunoglobulins, albumin, and the cellular housekeeping protein β‐actin in all group samples demonstrated that our separation method effectively removed contamination from the blood and cells (Figure 1I). These results indicated that we extracted EVs from the skin tissue, and a sub‐proportion study of total EVs found that TOM20^+^ MDVs specifically increased in the DFU group, suggesting that MDVs may potentially impact the progression of DFU.

### 2.2 Single‐Cell Sequencing Revealed Consistent Trends Between SNX9 and MDV in DFU

**Figure 2:**
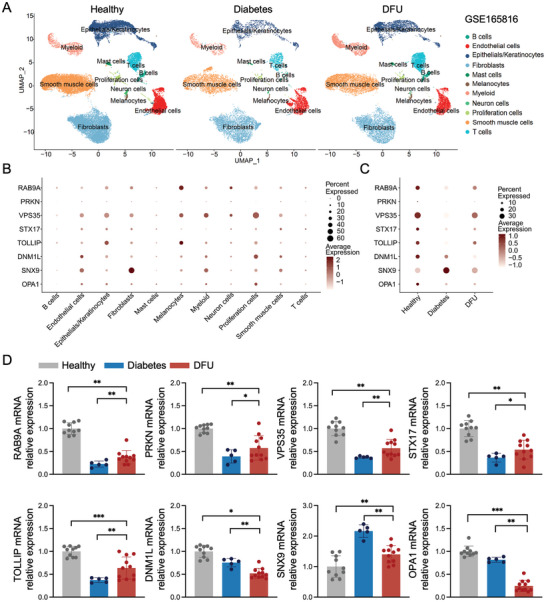
Expression levels of MDV‐related genes in single‐cell sequencing data. A) UMAP dimensionality reduction embedding of data from Healthy individuals, those with Diabetes, and those with DFU. B) Human single‐cell RNA sequencing dot plot demonstrating gene expression within various clusters; these clusters were identified using UMAP cluster analysis, as shown in (A). C) Human single‐cell RNA sequencing dot plot demonstrating gene expression within Healthy, Diabetes, and DFU samples. D) qRT‐PCR assay results (*RAB9A*, *PRKN*, *VPS35*, *STX17*, *TOLLIP*, *DNM1L*, *SNX9*, and *OPA1*). Statistical analysis was performed using one‐way ANOVA (Healthy, n = 3; Diabetes, n = 5; DFU, n = 5). Data are presented as the mean ± SD. *p < 0.05, **p < 0.01, ***p < 0.001, ****p < 0.0001.

We used the single‐cell sequencing dataset GSE165816, as reported by Theocharidis.^[^ ^34^ ^]^ This dataset includes categories similar to those used in our previous studies: healthy, diabetes, and DFU. Our analysis focused on the non‐healing DFU group from this dataset and excluded the healed DFU group. To map the transcriptome and cellular landscape of DFUs, we analyzed the single‐cell profiles of foot samples from the dataset GSE165816. Split Uniform Manifold Approximation and Projection (UMAP) analysis revealed the cell types in the three clinical groups (**Figure** **2**A).

We focused on the genes related to the biogenesis or secretion of MDV, including *CD38*, *RAB9A*, Parkin RBR E3 Ubiquitin Protein Ligase (*PRKN*), VPS35 Retromer Complex Component (*VPS35*), Syntaxin 17 (*STX17*), Toll Interacting Protein (*TOLLIP*), Dynamin 1 Like (*DNM1L*), *SNX9*, and OPA1 Mitochondrial Dynamin Like GTPnase (*OPA1*). Figure 2B shows the expression patterns of these mRNAs in various cell types. *RAB9A* and *VPS35* were highly expressed in nearly all cell types, whereas *TOLLIP*, *DNM1L*, and *SNX9* were expressed in only a few specific cell types. In contrast, *PRKN* and *OPA1* were generally expressed at lower levels. Given the uncertainty regarding the source cells of MDVs, we first focused on fibroblasts, the most important cell type for the growth and development of skin tissue. Among fibroblasts, *RAB9A*, *VPS35*, and *SNX9* exhibited the highest expression levels. However, when comparing between groups (Figure 2C), the expression levels of *RAB9A* and *VPS35* were higher in the healthy group than in the DFU group. *SNX9*, a gene with high fibroblast expression and higher expression in the DFU group, is believed to be closely related to MDV secretion.

We measured the protein concentration of MDVs extracted from fibroblasts under various conditions using the BCA method (Figure S2F, Supporting Information). Under normal glucose conditions, WT cells showed a baseline MDV protein concentration. SNX9 knockdown (SNX9‐KD) and SNX9 inhibiton (SNX9‐IN) exhibited significantly reduced MDV protein levels compared to those in the WT, whereas those in SNX9 overexpression (SNX9‐OE) were markedly increased. In high glucose (HG) conditions, cells exhibited a significantly higher MDV protein concentration (180.27 ± 6.14 µg mL^−1^) compared to that under normal glucose conditions. This increase was amplified in the SNX9‐OE group. Notably, the SNX9‐KD and SNX9‐IN groups exhibited attenuated HG‐induced increases in MDV protein concentration, bringing levels closer to those observed under normal glucose conditions. These results suggest that SNX9 plays a crucial role in regulating MDV secretion by fibroblasts, particularly under HG conditions. The inhibition or knockdown of SNX9 effectively reduced MDV secretion, whereas its overexpression enhanced secretion, especially in an HG environment. Furthermore, we analyzed the collected skin tissue (Figure 2D), and the changes in the expression levels of these genes were consistent with the trend observed in single‐cell sequencing. Therefore, in DFU, fibroblasts secrete a large amount of MDVs under the regulation of SNX9. However, how this leads to further disease development requires elucidation of the functions of MDVs in DFU. In this study, we conducted in vitro experiments using hgMDVs.

### 2.3 Fibroblast‐Derived MDVs Have Components Similar to Tissue MDVs

**Figure 3:**
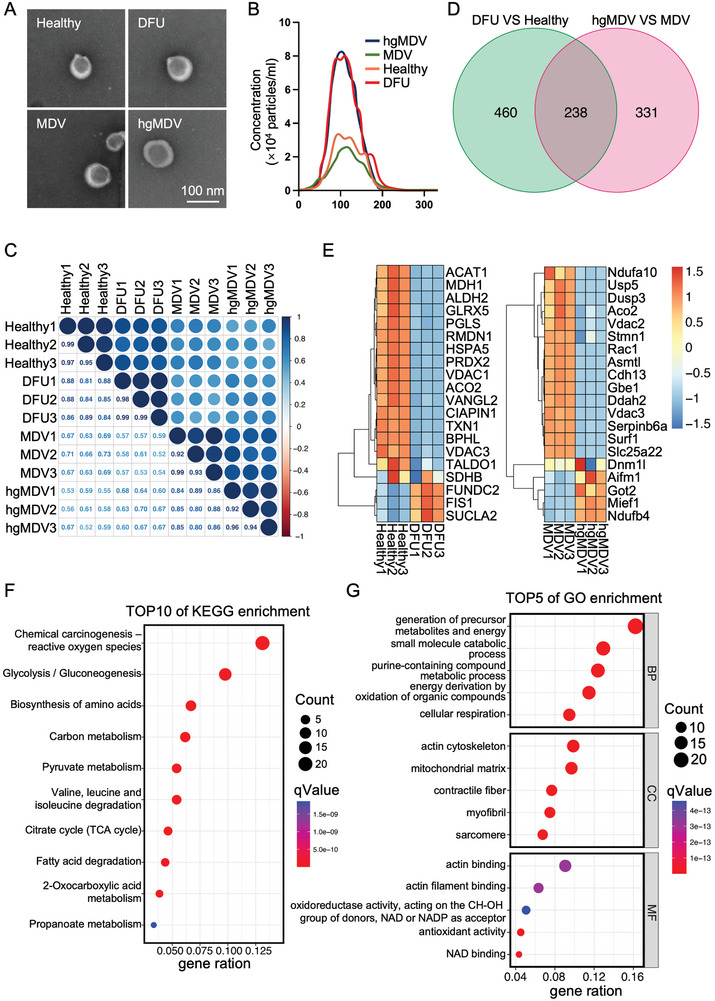
Proteomic profiling of MDVs. A) TEM images of MDVs from Healthy, DFU, MDV, and hgMDV groups. B) Size distribution of MDV particles. C) Pearson's correlation coefficients between protein profiles of MDVs from each group. D) The Venn diagram shows the intersections of DEPs between DFU versus Healthy groups and hgMDV versus MDV groups. E) Heatmap showing DEPs in MDVs between DFU versus Healthy groups and hgMDV versus MDV groups. F) KEGG pathway enrichment analysis of 238 common DEPs. G) GO annotation analysis of 238 common DEPs.

After culturing fibroblasts in the HG medium, MDVs were extracted from the cell culture medium and referred to as hgMDVs to distinguish them from MDVs extracted from the normal cell culture medium (referred to as MDVs). Transmission electron microscopy (TEM) analysis of purified MDVs from the Healthy and DFU groups, MDVs, and hgMDVs was performed. All MDVs exhibited typical membrane structures with diameters ranging from 60–130 nm (**Figure** **3**A). A wider field of view corresponding to this TEM image is shown in Figure S3 (Supporting Information). The NTA also demonstrated that the MDVs were within this particle size range. However, in terms of particle size, similar to the healthy group, fibroblasts in the normal medium also showed low levels of MDV secretion. Nevertheless, more MDVs were present in the skin tissue of the DFU groups and fibroblasts cultured in an HG environment, indicating that DFU and HG culture media promoted the secretion of MDV (Figure 3B). Next, to investigate the protein components of MDVs, we performed a 4D label‐free quantitative proteomics analysis and compared the MDVs in the Healthy and DFU groups and the hgMDV and MDV groups via correlation analysis. Correlation analysis revealed that these sub‐clusters had similar expression profiles (Figure 3C), demonstrating that the protein content of MDV in skin tissue and cell culture medium was similar. We compared the 2‐fold of differentially expressed proteins (DEPs) between the Healthy and DFU groups and the MDV and hgMDV groups. Based on the results of the two differential analyses, 238 proteins intersected (Figure 3D). The heatmap showed the top 20 DEPs for the two differential analyses (Figure 3E). Acetyl‐CoA Acetyltransferase 1 (ACAT1), Malate Dehydrogenase 1 (MDH1), Aconitase 2 (Aco2), and Solute Carrier Family 25 member 22 (Slc25a22) related to metabolism and mitochondrial homeostasis were expressed at lower levels in DFU and hgMDV, whereas SDHB, FUN14 Domain‐Containing 2 (FUNDC2), and Fission 1 (FIS1) related to oxidative stress were expressed at higher levels in both groups. Based on the 238 DEPs, we performed a Kyoto Encyclopedia of Genes and Genomes (KEGG) pathway enrichment analysis to understand the correlative pathways and Gene ontology (GO) annotation to understand the functional properties of different proteins. Chemical carcinogenesis, ROS, glycolysis/gluconeogenesis, and the citrate (TCA) cycle showed significant changes based on DEPs (Figure 3F). GO annotation analysis revealed that the DEPs correlated with biological processes, cellular components, and molecular functions. Cellular components were associated with mitochondrial components, and molecular functions were predominantly associated with oxidative stress and aerobic metabolism (Figure 3G). Based on the proteomic analysis of MDVs, it has been suggested that MDVs may regulate mitochondrial dynamics, respiratory metabolism, and oxidative stress. Furthermore, hgMDVs are expected to serve as functional substitutes for DFU in MDV‐related in vitro studies because of their high similarity to those found in DFUs.

### 2.4 hgMDVs Promotes Cell Apoptosis, Intracellular Oxidative Stress, Δψm Reduction, and Mitochondrial Structural Damage

**Figure 4:**
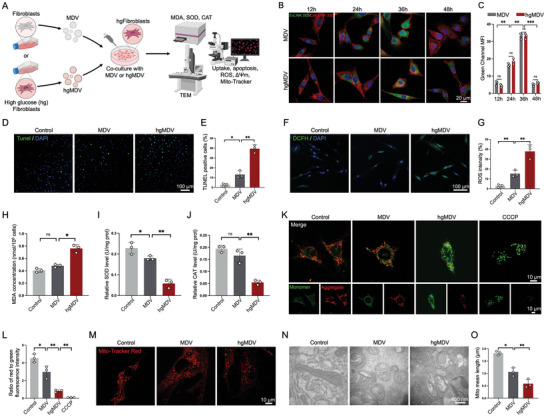
Effects of MDVs and hgMDVs on fibroblasts. A) Experimental design: Fibroblasts cultured in HG medium were co‐cultured with MDVs or hgMDVs and analyzed for various cellular processes. B,C) Confocal microscopy images and quantitative analysis of fluorescence intensity for EvLINK 505‐labeled MDV internalization by CellLINK 555‐labeled fibroblasts at 12, 24, 36, and 48 h. Statistical analysis was performed using an independent Student's t‐test (n = 3). D) Representative images of the TUNEL assay. E) Semiquantitative analysis of TUNEL‐positive cells. Statistical analysis was performed using one‐way ANOVA (n = 3). F) Representative fluorescence images showing ROS formation (DCFH‐DA) in fibroblasts after exposure to MDVs and hgMDVs. G) Quantitative analysis of ROS signals in fibroblasts. Statistical analysis was performed using one‐way ANOVA (n = 3). H) MDA levels in fibroblasts were quantitatively determined using an MDA assay kit. Statistical analysis was performed using one‐way ANOVA (n = 3). I) Effect of the indicated treatments on SOD activity in fibroblasts. Statistical analysis was performed using one‐way ANOVA (n = 3). J) Effect of the indicated treatments on CAT activity in fibroblasts. K) Effects of MDVs on mitochondrial membrane potential were determined using JC‐1 staining. L) Quantitative ratio analysis of aggregated and monomeric JC‐1 is shown in panel (n = 3). M) Representative confocal images showing mitochondrial morphology in fibroblasts. Cells were stained with MitoTracker Deep Red. N) TEM images of mitochondrial structure in fibroblasts. O) Quantitative analysis of mitochondrial mean length (n = 3). Data are presented as mean ± SD. Statistical analysis was performed using one‐way ANOVA. *p < 0.05, **p < 0.01, ***p < 0.001, ns: *p* > 0.05.

To investigate the effects of MDVs and hgMDVs on fibroblasts, we designed the experimental setup as illustrated in **Figure** **4**A. Fibroblasts were cultured under HG conditions (serving as the Control group) and then co‐cultured with either MDVs or hgMDVs. To clarify the internalization efficiency of MDVs by cells, we performed EvLINK 505 labeling of MDVs and detected co‐cultured CellLINK 555‐labeled fibroblasts at different time points. After 36 h of incubation, several MDVs were internalized into the cytoplasm and exhibited high‐intensity green fluorescence. However, after 48 h, a significant decrease in green fluorescence intensity was observed (*p* < 0.001). There was no significant difference in fluorescence intensity between the MDV and hgMDV groups simultaneously. Nonetheless, the fluorescence intensities between two adjacent time points differed significantly (*p* < 0.05). These results indicate that the internalization efficiency of MDV and hgMDV by fibroblasts peaked at 36 h and then declined (Figure 4B,C). TUNEL staining was performed to evaluate the apoptosis levels in all groups. As depicted in Figure 4D,E, after treatment with hgMDV, the number of TUNEL‐positive cells significantly increased compared with that in the MDV group. These results indicate that hgMDV treatment significantly promotes fibroblast apoptosis. A DCFH‐DA fluorescent probe was used to assess the intracellular ROS levels (Figure 4F,G). ROS levels in the hgMDV group were significantly higher than those in the control and MDV groups. A similar trend was observed for MDA, the end product of lipid peroxidation (Figure 4H). Furthermore, hgMDV treatment decreased the antioxidant effects by reducing the levels of SOD and CAT (Figure 4I,J). These results indicate that hgMDV promotes apoptosis and increases intracellular oxidative stress in fibroblasts, consistent with the phenotype of fibroblasts in DFU.

In Figure 4K, the mitochondrial membrance potential (Δψm) was measured using the JC‐1 probe, revealing that the control and MDV groups had higher Δψm, allowing the probe to enter mitochondria and exhibit aggregate fluorescence, while hgMDV significantly reduced Δψm in fibroblasts. CCCP, a mitochondrial depolarization inducer, causes JC‐1 to exist mostly in its monomeric form. The ratio of fluorescence under 488 and 594 nm laser channels can quantify the level of Δψm; the control group was 2‐fold and 4‐fold higher than that in the MDV and hgMDV groups, respectively (*p* < 0.05) (Figure 4L). Confocal images of MitoTracker‐Red‐labeled mitochondria were used to observe mitochondrial morphology. The hgMDV group had smaller mitochondria and exhibited a more punctiform structure with a significant reduction in length, in stark contrast to the predominantly filamentous morphology observed in the control and MDV groups. This suggests that hgMDV changed the morphology of the mitochondria in fibroblasts, making them more independent, shrinking, and damaging the mitochondrial network (Figure 4M). TEM analysis (Figure 4N) was conducted to investigate alterations in mitochondrial ultrastructure. Mitochondria in the control group displayed a bilayer membrane and well‐organized cristae with tight stacking, while those in the MDV group exhibited swelling, along with a swollen and unclear inner membrane with fragmentation. Damage to the mitochondrial ultrastructure significantly increased after treatment with hgMDV, leading to a reduction in or the absence of mitochondrial cristae. The mitochondrial length in the hgMDV group was approximately one‐third that of the control group (Figure 4O). The results show that hgMDV induces damage to mitochondrial structure and Δψm, suggesting that changes in metabolic function may have already occurred in cells.

### 2.5 hgMDV Induction Suppresses the Level of Aerobic Metabolism

**Figure 5:**
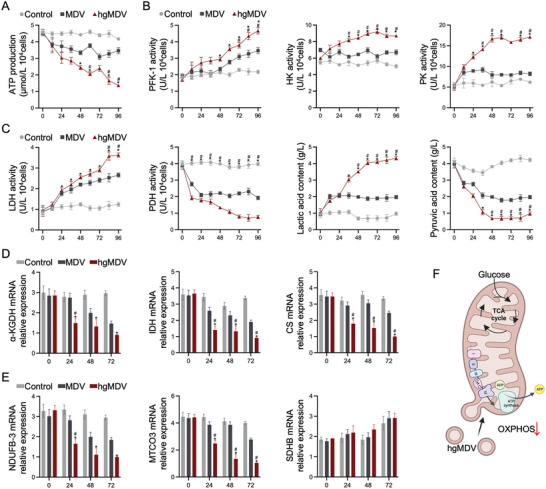
hgMDV induction suppresses aerobic metabolism. A) ATP production was measured at different time points post‐treatment (n = 3). B) PFK‐1, HK, and PK activities were measured at different time points after treatment (n = 3). C) Activities of LDH and PDH and concentrations of lactic and pyruvic acids were measured (n = 3). D) Relative quantitative analysis was performed on the expressions of *𝛼‐KGDH*, *IDH*, and *CS* (n = 3). E) Relative quantitative analysis was performed on expressions of *NDUFB‐3*, *MTCO3*, and *SDHB* (n = 3). F) Schematic diagram of the role of hgMDVs on mitochondrial OXPHOS. Data are presented as mean ± SD. Statistical analysis was performed using one‐way ANOVA. *: *p* < 0.05 with respect to the Control group, #: *p* < 0.05 with respect to the MDV group.

On the third day following induction with MDV and hgMDV, various aspects of glycometabolism were assessed in the control, MDV, and hgMDV groups. Assessments included analysis of glycolytic enzyme activity, mRNA levels of tricarboxylic acid cycle enzymes, and mRNA levels of the oxidative phosphorylation (OXPHOS) complex subunits. As shown in **Figure** **5**A, compared with the control group, Adenosine Triphosphate (ATP) production significantly decreased in the MDV and hgMDV groups following treatment, with the hgMDV group exhibiting the lowest ATP production. Figure 5B shows the activities of glycolytic rate‐limiting enzymes, such as 6‐Phosphofructokinase‐1 (PFK‐1), Pyruvate Kinase (PK) and Hexokinase (HK). Preliminary findings indicated that hgMDV treatment increased the reliance of fibroblasts on glycolytic function. Enzyme activity in the hgMDV group increased within 72 h, reaching a significantly higher level than the control group (*p* < 0.05). The Lactate Dehydrogenase (LDH) and PDH activities were also examined. LDH activity increased in the hgMDV group, whereas PDH activity decreased during treatment. Lactic acid content in the hgMDV group increased, whereas the pyruvic acid content decreased (Figure 5C). The mRNA expression levels of key enzymes involved in the tricarboxylic acid cycle, including Alpha‐Ketoglutarate Dehydrogenase (*α‐KGDH*), Isocitrate Dehydrogenase (*IDH*), and Citrate Synthase (*CS*), were examined using RT‐qPCR (Figure 5D), which showed downregulation of *α‐KGDH*, *IDH*, and *CS* expression in the hgMDV group. Notably, the expression levels of *α‐KGDH*, *IDH*, and *CS* in the control and MDV groups were significantly different from those in the hgMDV group after 24 h; the downregulation observed in the hgMDV group was the most significant compared with the MDV group (*p* < 0.05). Figure 5E shows the mRNA expression levels of NADH Dehydrogenase 1‐β3 (*NDUFB3*), Mitochondrial Encoded Cytochrome C Oxidase III (*MTCO3*), and Succinate Dehydrogenase Complex Iron Sulfur Subunit B (*SDHB*) determined using RT‐qPCR. The expression levels of *NDUFB3* and *MTCO3* in the control and MDV groups differed significantly from those in the hgMDV group after 24 h (*p* < 0.05), whereas *SDHB* expression did not differ among the three groups (*p* > 0.05). These findings indicated a significant impairment of mitochondrial respiratory function in the hgMDV group (Figure 5F).

### 2.6 Impact of SNX9 Knockdown, Inhibition, and Overexpression on Expression and MDV Levels

Our previous study utilizing single‐cell sequencing, we identified SNX9 as a potential molecule affecting MDV secretion in DFU. To elucidate the role of SNX9 in MDV secretion, we generated SNX9‐KD, SNX9‐IN, and SNX9‐OE fibroblasts and measured MDV production in both normal and HG media (Figure S2A, Supporting Information). The expression level of SNX9 was significantly reduced by siRNA. However, the SNX9 inhibitor DATPT did not notably decrease the content of SNX9, likely because this inhibitor primarily functions by blocking the interaction between SNX9 and p47phox, rather than directly downregulating SNX9 expression. Additionally, the overexpression plasmid significantly elevated the expression levels of SNX9. These trends were consistent in both normal and HG environments, although the expression levels of SNX9 were slightly higher in HG conditions than in normal fibroblasts (Figure S2B,C, Supporting Information).

Using the same differential centrifugation and immunoprecipitation methods as previously described, we isolated MDVs. NTA revealed that siRNA or inhibitor treatment significantly reduced MDV concentrations, whereas SNX9 overexpression significantly increased MDV concentrations. Notably, the concentration of MDVs was elevated under HG conditions (Figure S2D, Supporting Information). Neither SNX9 levels nor the HG environment significantly altered the morphology or size of the MDVs (Figure S2E, Supporting Information). By measuring the protein concentration of MDVs in each group using the BCA method, we found that SNX9‐KD and SNX9‐IN significantly reduced the MDV protein content, whereas SNX9‐OE had the opposite effect (Figure S2F, Supporting Information). Therefore, there was a positive correlation between MDV production, SNX9 protein levels, and the interaction between SNX9‐p47phox (the target of the SNX9 inhibitor). This experiment confirmed the in vitro hypothesis that SNX9 promotes MDV secretion under HG conditions.

### 2.7 hgMDVs Impairs Wound Healing in a Full‐Thickness Cutaneous Wound Model of Diabetes In Vivo

**Figure 6:**
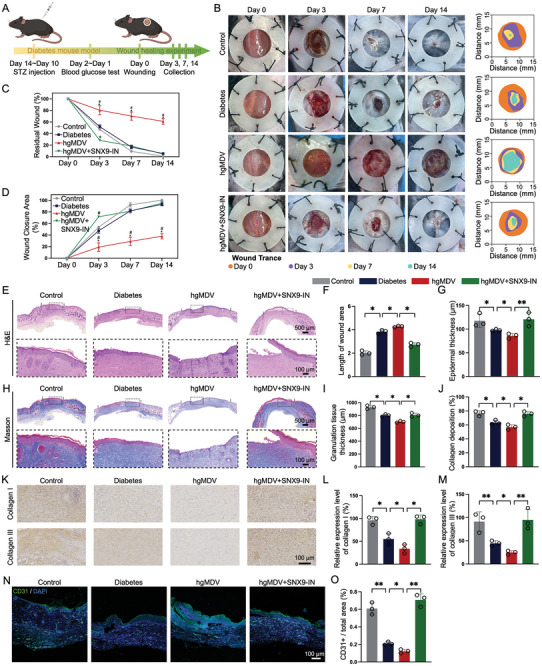
Histological analysis of diabetic wound healing modulated by hgMDVs and SNX9 inhibition. A) Schematic illustration of the operations performed on mice, arranged in chronological order. B) Representative images of wounds on days 0, 3, 7, and 14 in Control, Diabetes, hgMDV, and hgMDV+SNX9‐IN groups and diagrams of time‐evolved wound areas. C) Percentage of residual wounds at days 0, 3, 7, and 14 in each group (n = 3). *: *p* < 0.05 with respect to hgMDV+SNX9‐IN group; #: *p* < 0.05 with respect to the Diabetes group. D) Percentage of wound closure area at days 0, 3, 7, and 14 in each group (n = 3). *: *p* < 0.05 for hgMDV+SNX9‐IN group; #: *p* < 0.05 for Diabetes group. E) Hematoxylin and eosin staining of the collected skin tissue at day 14 post‐wounding. F) Quantitative analysis of wound area length (n = 3). G) Quantitative analysis of epidermal thickness (n = 3). H) Masson's trichrome staining of the collected skin tissue 14 days post‐wounding. I) Quantitative analysis of granulation tissue thickness within wound area (n = 3). J) Quantitative analysis of the collagen deposition in wound center area (n = 3). K) Collagen I and III immunohistochemical staining in each group on day 7. L,M) Quantitative data for collagen I and III in skin tissues (n = 3). (N) Immunofluorescence staining of CD31 in granulation tissues in each group. O) Quantitative analysis of CD31‐positive area (n = 3). Data are presented as the mean ± SD. Statistical analysis was performed using one‐way ANOVA. *p < 0.05, **p < 0.01.

The in vivo wound‐healing performance of hgMDVs was evaluated in a full‐thickness cutaneous wound model in diabetic mice (**Figure** **6**A). All the mice in the four groups underwent surgical modeling of 10 mm full‐thickness cutaneous wounds. The Control group comprised normal mice, whereas the diabetes group comprised diabetic mice treated with saline drops. The hgMDV group and hgMDV+SNX9‐IN groups consisted of diabetic mice treated as follows: the hgMDV group received 25 µL of 60 µg mL^−1^ hgMDV solution. The hgMDV+SNX9‐IN group received a combination of 25 µL of 60 µg mL^−1^ hgMDVs and the SNX9 inhibitor. The SNX9 inhibitor was administered at a concentration of 800 µg mL^−1^ with a volume of 1.25 µL g^−1^ of body weight.

Wound healing was observed and recorded on days 3, 7, and 14. On day 3 after wound formation, the hgMDV group had the lowest wound closure area percentage of 19.1 ± 1.2%. The hgMDV+SNX9‐IN group exhibited enhanced wound healing, with a decrease in the residual wound area of 51.1 ± 4.6% and 23.6 ± 3.4% compared with those in the hgMDV and diabetes groups, respectively. On day 14, the wound area visibly shrank in all groups, and the differences between these groups were further emphasized. Notably, the hgMDV group received the least effective therapy, with a healing rate of 37.9%. The SNX9 inhibitor and diabetes groups had similar percentages of closed wound areas (95.1% and 93.0%, respectively). By day 14, the control group showed almost no skin defects (Figure 6B–D). However, wound regions were still observed in diabetes, hgMDV, and hgMDV+SNX9‐IN groups, constituting 5.3%, 61.1%, and 4.6% of the original wound area, respectively. These results indicated that hgMDVs significantly slowed wound healing, whereas the SNX9 inhibitor promoted diabetic wound healing.

On day 14, the wound tissue samples from each group were collected for H&E staining (Figure 6E). Wound lengths were measured (Figure 6F). Histological assessment primarily targets the overall morphology of the wound bed with a specific focus on the regeneration of epidermal and granulation tissues, given that the processes of re‐epithelialization and granulation tissue formation are recognized as key indicators of wound healing.^[^ ^35^ ^]^ By day 14, neo‐epidermis formation occurred in the hgMDV+SNX9‐IN group, with the thickest neo‐epidermis measuring 120.6 ± 17.3 µm (Figure 6G). Collagen deposition, crucial for skin remodeling and enhancing tissue tensile strength and epidermal integrity, was evaluated in the wound bed using Masson's trichrome staining (Figure 6H). Treatment with the SNX9 inhibitor resulted in denser granulation tissue than in the diabetes group. More compact granulation tissue was formed in wounds treated with the SNX9 inhibitor, which was thicker than that observed in the hgMDV groups (Figure 6I). On day 14, the hgMDV+SNX9‐IN group showed significantly more collagen deposition in the regenerated tissue than the diabetes and hgMDV groups, highlighting the effective role of the SNX9 inhibitor in this process (Figure 6J). Collagen types I and III, as primary components of the dermal ECM, promote diabetic wound healing and remodeling.^[^ ^36^ ^]^ Immunohistochemical analysis revealed that wounds treated with the SNX9 inhibitor exhibited increased production of collagens I and III, which suggests that more mature collagen was formed in the hgMDV+SNX9‐IN group than in the Diabetes and hgMDV groups (Figure 6K–M). Collagen I and III immunohistochemistry results were consistent with those of Masson's staining, showing ample and well‐organized collagen fibers in the SNX9 inhibitor‐treated groups, indicating higher levels of collagen deposition than in the Diabetes and hgMDV groups, which promotes wound healing and improves tissue tensile strength.

Neovascularization, which is critical for diabetic wound healing due to the regenerated tissue's reliance on oxygen and nutrients from blood vessels, was evaluated using CD31, a marker for neovascularization. At day 14, very limited blood vessel structures were detected in both the Diabetes and hgMDV groups (Figure 6N,O). In contrast, treatment with the SNX9 inhibitor led to significantly enhanced neovascularization, characterized by more extensive and complete blood vessel formation with well‐defined luminal structures, making it the most effective among the tested groups. The enhanced angiogenesis mediated by the SNX9 inhibitor facilitated skin tissue regeneration during the diabetic wound healing process, yielding beneficial therapeutic outcomes. In summary, the SNX9 inhibitor promotes diabetic wound healing by enhancing collagen deposition and angiogenesis.

### 2.8 Role of hgMDVs in Delayed Diabetic Wound Healing

**Figure 7:**
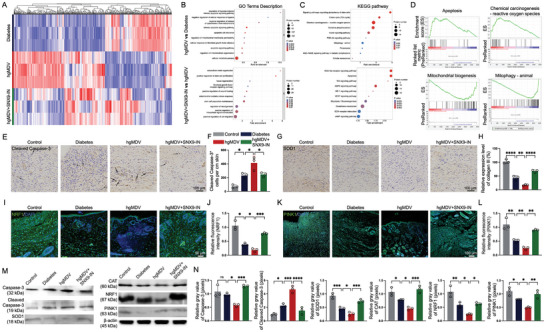
Analysis of molecular pathways and protein expression in diabetic wound healing under hgMDV treatment. A) Heatmap showing differential gene expression across control, diabetes, and hgMDV groups (n = 3). B) GO term enrichment analysis: upper panel shows hgMDV versus diabetes groups, and lower panel shows hgMDV+SNX9‐IN versus hgMDV groups. C) KEGG pathway enrichment analysis: upper panel shows hgMDV versus diabetes groups, and lower panel shows hgMDV+SNX9‐IN versus hgMDV group. D) GSEA plots for key pathways in hgMDV versus diabetes group comparison: apoptosis, chemical carcinogenesis–reactive oxygen species, mitochondrial biogenesis, and mitophagy. E–H) Immunohistochemistry and quantification of Cleaved Caspase‐3 and SOD1 expression in wound tissues (n = 3). I–L) Immunofluorescence staining and quantification of NRF1 and PINK1 in wound tissues (n = 3). M) Western blot analysis of key proteins involved in apoptosis, mitochondrial function, and oxidative stress. N) Quantification of protein expression levels from western blot analysis (n = 3). Scale bars: 100 µm. Data are presented as mean ± SD. Statistical analysis was performed using one‐way ANOVA. *p < 0.05, **p < 0.01, ***p < 0.001, ****p < 0.0001.

To explore the molecular mechanisms underlying the delayed healing of diabetic wounds caused by MDVs, we conducted a comprehensive proteomic analysis of wound tissues from diabetic mice following hgMDV intervention. Our analysis revealed significant differences in protein expression and pathway activation between hgMDV‐treated and diabetic conditions and between hgMDV treatment with and without SNX9 inhibition. The heatmap and hierarchical clustering (**Figure** **7**A) revealed distinct protein expression patterns among the Diabetes, hgMDV, and hgMDV+SNX9‐IN groups. By comparing the hgMDV‐treated and diabetic conditions, we identified 174 upregulated and 99 downregulated proteins. When examining the effects of SNX9 inhibition on hgMDV treatment, we observed 60 upregulated and 93 downregulated proteins.

GO and KEGG pathway analyses (Figure 7B,C) identified significantly enriched biological processes and pathways affected by hgMDV treatment and SNX9 inhibition, including mitochondrial dynamics, oxidative stress response, and apoptosis. Further analysis of GO terms revealed that hgMDV treatment, compared to diabetic conditions alone, significantly affected processes related to hypoxia‐induced apoptosis, mitochondrial function, and cellular metabolic processes. Specifically, we observed enrichment in terms of regulating hypoxia‐induced intrinsic apoptotic signaling, negative regulation of cellular response to hypoxia, and regulation of mitochondrial membrane permeability. These results indicate that hgMDVs may impair wound healing by disrupting cellular responses to hypoxic conditions and mitochondrial homeostasis. Interestingly, the addition of SNX9 inhibitors (SNX9‐IN) mitigated many of the detrimental effects of hgMDV.

KEGG analysis comparing hgMDV+SNX9‐IN to hgMDVs alone revealed enrichment in NOD‐like receptor signaling, apoptosis, Wnt signaling, and glutathione metabolism pathways. GO term analysis supported this, showing enrichment in processes related to extracellular matrix organization, stem cell proliferation, tissue regeneration, and positive regulation of wound healing. Notably, in the KEGG pathway analysis of the hgMDV versus diabetes groups, we observed significant enrichment in pathways related to stem cell pluripotency regulation, the TCA cycle, ROS, OXPHOS, insulin signaling, PI3K‐Akt signaling, mitophagy, peroxisome function, AGE‐RAGE signaling, and cellular senescence.

Gene Set Enrichment Analysis (GSEA) comparing hgMDVs to diabetes conditions revealed significant alterations in several key cellular pathways (Figure 7D). The analysis highlighted a strong positive enrichment in the apoptosis pathway, indicating a significant upregulation of genes associated with programmed cell death in the hgMDV group. Similarly, the chemical carcinogenesis‐ROS pathway showed positive enrichment, suggesting increased oxidative stress and ROS production under hgMDV treatment. Intriguingly, the mitochondrial biogenesis pathway and mitophagy pathways exhibited clear negative enrichment, indicating a significant downregulation of mitochondrial autophagy genes in the hgMDV group. These GSEA results collectively suggest that hgMDV treatment, compared with diabetic conditions alone, enhances apoptotic signaling, increases oxidative stress, and suppresses mitochondrial biogenesis and mitophagy.

Cleaved caspase‐3, a marker of apoptosis, was markedly increased in hgMDV‐treated wounds compared to diabetic controls, indicating enhanced apoptotic activity, with SNX9 inhibition partially reversing this effect (Figure 7E,F). SOD1, a crucial antioxidant enzyme,^[^ ^37^ ^]^ showed significantly decreased expression under diabetic conditions, which was further reduced upon hgMDV treatment. This suggests an exacerbation of oxidative stress by hgMDVs (Figure 7G,H). Nuclear Respiratory Factor 1 (NRF1), a key regulator of mitochondrial biogenesis, was downregulated under diabetic and hgMDV conditions, and SNX9 inhibition partially restored its expression (Figure 7I,J). Conversely, PTEN Induced Kinase 1 (PINK1), a mediator of mitophagy, was upregulated in hgMDV‐treated wounds and normalized by SNX9 inhibition (Figure 7K,L). Western blot analysis (Figure 7M,N) confirmed these protein expression changes, showing alterations in caspase‐3, cleaved caspase‐3, SOD1, CAT, NRF1, and PINK1 levels under different treatment conditions. SNX9‐IN treatment partially reversed the hgMDV‐induced increase in cleaved caspase‐3 and decrease in antioxidant enzymes (SOD1 and CAT) and mitochondrial function‐related proteins (NRF1 and PINK1). These results demonstrate that hgMDVs exacerbate diabetic wound healing by disrupting mitochondrial homeostasis, increasing oxidative stress, and promoting apoptosis and that SNX9 inhibition partially mitigates these effects. These results suggest that hgMDVs delay diabetic wound healing through multiple mechanisms, including exacerbation of oxidative stress, promotion of apoptosis, disruption of mitochondrial function, and impairment of cellular regenerative processes. SNX9 inhibition ameliorates these effects.

## 3 Discussion

Herein, we extracted EVs from skin tissue and sorted them using nanoflow technology, revealing an increase in MDV content. Consistent with previous studies,^[^ ^38^ ^]^ protein chip assays identified TOM20 as a marker, indicating an increase in MDV content. Proteomic sequencing was performed on MDVs extracted from both tissue and cell culture fluid supernatants. Our findings revealed a significant similarity between the two sources of MDVs, providing a solid foundation for future research. Genes associated with MDV production were highly expressed in patients with diabetic ulcers from the dataset GSE165816, with a similar expression trend observed in the tissues. MDVs, which have been recently recognized, are membranous structures secreted by mitochondria encompassing specific mitochondrial contents and ranging in size from 60 to 150 nm.^[^ ^10^ ^]^ Biochemical assays reveal their capability to recycle damaged and oxidized mitochondrial proteins without impairing the function of an individual mitochondrion,^[^ ^11^ ^]^ highlighting their crucial role in mitochondrial homeostasis. However, their physiological role has remained elusive. SNX9 serves as the adaptor protein that is necessary for the formation of MDVs on mitochondria.^[^ ^21^ ^]^ Previous studies have confirmed that SNX9 is associated with the formation of MDVs in immune cells.^[^ ^21^ ^]^ The high expression of SNX9 in DFU patients is likely to be the reason for the accompanying high MDV content.

The intricate regulation of multiple cells underlies the progression of DFU, with intercellular communication potentially mediated by EVs.^[^ ^39^ ^]^ In the skin tissue of the DFU group, we found a significant increase in MDVs, suggesting that these small EVs play a pivotal role in its pathogenesis. Despite limited research on MDVs in the context of DFU, their primary intracellular functions of mitochondrial quality control^[^ ^40^ ^]^ and antigen presentation^[^ ^21^ ^]^ have been well established. However, recent studies have highlighted their importance in intercellular communication.^[^ ^18^, ^40^ ^]^ Using nanoflow cytometry, we were able to distinguish subpopulations of TOM20^+^ EVs. In the diabetic tissues that had not yet progressed to DFU, there was a notable upregulation of PDH expression. This finding may implicate a subset of MDV‐containing mitochondrial matrix components. However, our study did not differentiate between TOM20^+^ MDVs and PDH^+^ MDVs, as they may have different functions and biogenesis pathways. Although the mechanisms underlying the secretion of MDVs remain unclear, a study by D'Acunzo et al. indicated that mitovesicles can be released into the extracellular space without being encapsulated by cellular membranes, which highlights the complexity of various secretion pathways and their functional implications. This finding suggests the feasibility of TOM20 immunoadsorption for isolating MDVs. However, it cannot be ruled out whether extracellular MDVs will be further encapsulated by plasma membrane components, nor can it be denied that TOM20⁻ PDH^+^ MDVs exist in extracellular space, resulting in omissions in the MDVs isolation (Figure S4, Supporting Information). Additionally, further research in this field may reveal how MDVs are released into the extracellular space, especially the interaction mechanism between MDVs and multivesicular bodies, helping us understand the significance of extracellular MDVs.

We detected larger vesicle sizes using NTA, which we attributed to vesicle aggregation, particularly considering the artifacts associated with ultracentrifugation. To obtain a more accurate size distribution in future studies, techniques such as tunable resistive pulse sensing and size‐sorting flow cytometry should be considered. Tunable resistive pulse sensing offers single‐vesicle resolution, minimizing aggregation artifacts, while size‐sorting flow cytometry allows size‐based separation and analysis of subpopulations, thus providing a more comprehensive characterization of the extracellular vesicle profile. The isolation of MDVs from tissue samples may be influenced by cellular damage, potentially resulting in the release of MDVs and mitochondrial membrane fragments. However, our application of a gentle enzymatic digestion method significantly mitigates this technical limitation, thereby better preserving the integrity of cell membranes and organelles. To investigate MDV further in vitro, we identified a model system utilizing fibroblasts. These cell‐derived MDVs have similar compositions and a high correlation to tissue‐derived MDVs. Cluster analysis allowed us to speculate on the potential roles of MDVs in oxidative stress and aerobic glycolysis. Notably, the secretion levels of MDVs in fibroblasts and skin tissue exhibited similar patterns. Healthy individuals exhibited low levels of MDV secretion, which necessitated large‐scale cell culture to obtain sufficient MDVs. This also made it challenging to consistently extract MDVs from clinical specimens for research purposes. Leveraging the single‐cell sequencing dataset from Georgios Theocharidis et al.,^[^ ^34^ ^]^ we unearthed further evidence supporting fibroblast‐derived MDV upregulation in DFU. Moreover, our findings implicate *SNX9* as a key gene promoting MDV secretion. However, contrasting results were obtained in a study by Heyn et al.,^18^ ^]^ where knocking down *SNX9* did not affect TOM20^+^ MDV secretion. Despite this, the use of SNX9 inhibitors reduced MDV production, indicating a contradictory conclusion. This discrepancy could be attributed to the use of distinct cell models and potential variations in MDV secretion mechanisms among different pathological conditions.

In the evolving field of extracellular vesicle research, the nomenclature for MDVs released into the extracellular space remains unclear. While the term “MDV” has been widely adopted in the literature, this terminology may not fully capture the complexity of these extracellular entities. We used the term “MDV” in this study because it remains the most widely recognized term in the field.^[^ ^32^, ^41^ ^]^ Specific genes play crucial roles in regulating MDV formation and secretion pathways. These genes are instrumental in controlling various aspects of the process, including MDV biogenesis, maturation, and release.^[^ ^14^ ^]^ The involvement of CD38 in intracellular calcium signaling affects mitochondrial function and dynamics,^[^ ^42^ ^]^ indirectly affecting the formation and function of MDVs. Furthermore, the silencing of RAB9A, a small GTPase, attenuates mitochondrial antigen presentation, indicating its critical involvement in the trafficking of MDVs.^[^ ^21^ ^]^ Parkin (encoded by the PRKN gene) is essential for initiating the degradation of damaged mitochondria through mitophagy, a process that targets entire mitochondria, or by inducing the formation of MDVs, which target damaged proteins and lipids for degradation in lysosomes.^[^ ^43^ ^]^ Additionally, VPS35, a vacuolar protein sorting‐associated protein, is necessary for the trafficking of MDVs to peroxisomes.^[^ ^44^ ^]^ The targeting of MDVs to lysosomes involves STX17, a SNARE protein essential for driving membrane fusion processes in a complex with other members of the SNARE family. Silencing STX17 has been shown to reduce the number of MDVs that are directed to lysosomes.^[^ ^45^ ^]^ TOLLIP plays a key role in Parkin‐dependent endosomal transport and aids in routing a particular group of MDVs toward lysosomes for delivery.^[^ ^38^ ^]^ DNM1L, also known as Drp1, is a GTPase involved in regulating mitochondrial and MDV division.^[^ ^11^ ^]^ SNX9, a sorting nexin essential for endosomal trafficking, is recruited to the mitochondrial surface to facilitate the formation of MDVs containing matrix proteins.^[^ ^41^ ^]^ Matheoud et al. identified SNX9 as a crucial component in MDV formation. They demonstrated that MDV formation depends on SNX9 and requires the recruitment of SNX9 and Rab9 to the mitochondria. Furthermore, silencing SNX9 inhibits MDV formation induced by oxidative stress.^[^ ^21^ ^]^ OPA1, a protein similar to dynamin that acts as a GTPase, is found in the inner membrane of mitochondria, where it helps mitochondria fuse together.^[^ ^46^ ^]^ OPA1 is essential for forming matrix‐positive MDVs,^[^ ^41^ ^]^ and SNX9 plays a crucial role in forming inflammation‐associated matrix‐positive MDVs.^[^ ^47^ ^]^

We have highlighted the progress in exploring the mechanisms underlying MDV formation; however, new challenges have emerged. Many proteins identified to date perform additional cellular functions. This complicates the interpretation of any loss‐of‐function experiments, as the observed effects may not solely be related to a reduction in MDVs but could also involve disruptions in mitochondrial and peroxisomal fission (DRP1) or indirect effects on endocytosis (SNX9) and the endomembrane system (VPS35). These challenges remain until MDV‐specific protein machineries have been identified.^[^ ^10^ ^]^

SNX9 Inhibitor Information: The SNX9 inhibitor used in our study was DATPT (MedChemExpress, HY‐145307). Lee et al. identified DATPT as a _12_WLVSKF_17_ peptide‐mimetic small molecule. DATPT can block SNX9−p47phox interactions in the endosome and suppress ROS and inflammatory cytokine production. In addition, the biological safety of DATPT was preliminarily investigated. Lee et al. demonstrated that DATPT exhibits high potency with an IC50 of 10 nM while maintaining good biocompatibility and low cytotoxicity in vitro. The compound displayed excellent stability in human plasma and liver microsomes, with 100% and 93% retention, respectively, after 30 min. Furthermore, DATPT showed no significant inhibition of the major cytochrome P450 isozymes.^[^ ^48^ ^]^ Our findings demonstrate a reduction in MDV production following treatment with the SNX9 inhibitor DATPT. However, in this study, we did not design our experiments to observe the dose‐effect relationship between hgMDV and the SNX9 inhibitor, the inhibitor concentration reported in previous studies was used. This restricts the strength of our conclusions regarding the specific role of SNX9 in MDV production and the therapeutic potential of DATPT. Determining the lowest effective concentration, dose dependent effects and threshold effects of the SNX9 inhibitor to reduce MDV formation would indeed be valuable for future research.

To date, no factors specific to MDV formation have been identified, limiting our comprehension of MDV's contributions to DFU. Considering that MDVs and exosomes^[^ ^49^ ^]^ share similar membranous structures and sizes, we drew inspiration from exosome research to design our experiments to analyze treatment with exogenous MDVs. As anticipated, the fibroblasts were able to take up these external MDVs. Cellular experiments demonstrated that MDVs can be successfully internalized by cells, leading to increased apoptosis, oxidative stress, mitochondrial damage, and glycolysis. Mitochondrial function assays confirmed that the MDV‐mediated effects were mitochondrial‐focused, with MDV causing a significant reduction in aerobic respiration and Δψm. Together, these results highlight a role for MDVs in mitochondrial homeostasis. In the analysis of oxidative stress, glycolysis, and glucose metabolism, we found that the mitochondria in cells treated with hgMDV exhibited high oxidative stress and high glycolytic activity. Given the significant role that MDVs play, further studies are needed to reveal the responsiveness of mitochondria to oxidative stress by forming MDVs.

Increasing evidence indicates that MDVs are critical for immune and metabolic responses, playing a key role in maintaining tissue and organ homeostasis.^[^ ^50^ ^]^ These vesicles can form under both basal conditions and mild oxidative stress, effectively eliminating oxidized proteins in various cellular states, both normal and pathological.^[^ ^18^, ^19^ ^]^ MDVs exhibit clear cargo specificity under different stress conditions. Specifically, during oxidative stress, the enriched proteins predominantly come from substrates involved in ATP synthesis that have been oxidized.^[^ ^14^ ^]^ Indeed, MDVs operate as a dynamic balance system in healthy mitochondria, ensuring the regular clearance of oxidized components.^[^ ^51^ ^]^ However, when mitochondrial self‐renewal pathways fail, the clearance of dysfunctional organelles via MDVs can release harmful material, potentially triggering inflammation.^[^ ^52^ ^]^ Furthermore, the release of MDVs, which contain mtDNA, can lead to cytokine activation and a stronger inflammatory response, as observed in cells treated with fumarate.^[^ ^32^ ^]^ These studies have illustrated the importance of MDVs as a vital pathway for the transfer of mitochondrial components influencing cellular metabolism. We validated the MDV‐enriched pathway highlighted in previous proteomics studies affecting cellular functions and noted that MDVs in an HG culture environment promote apoptosis and ROS generation. Although we observed this phenomenon, we did not explore the mechanism in depth. Our initial hypothesis was that mitochondrial‐related components within MDVs, such as mitochondrial DNA or cytochrome C, mediate apoptosis or oxidative stress processes. Furthermore, focusing on how MDVs affect mitochondria, we noted alterations in Δψm, size, and structure, including shortening, shrinking, granularity, and vacuole‐like damages in the ultrastructure. These observations are consistent with the findings of Guo et al.,^[^ ^25^ ^]^ affirming the damaging effects of MDVs on mitochondria. Further, we measured the levels of glucose metabolism and found that MDV promoted glycolysis but reduced aerobic respiration, except for the SDHB complex, whose function in the electron transport chain (ETC) does not directly involve molecular oxygen, a finding consistent with mitochondrial damage. According to previous studies, an over‐reliance on glycolysis may further aggravate cellular mitochondrial dysfunction, potentially triggering multiple apoptotic pathways. Glycolysis can decrease intracellular pH and potentially lead to the opening of the mitochondrial permeability transition pore; however, these pathways have yet to be experimentally confirmed.

Mitochondrial dynamics, including the balance between fusion and fission processes, are crucial for maintaining mitochondrial function and homeostasis.^[^ ^53^ ^]^ The Δψm, maintained by OXPHOS, is essential for various mitochondrial functions.^[^ ^54^, ^55^ ^]^ In our study, MDVs under conditions of hyperglycemia disrupted mitochondrial dynamics and homeostasis by reducing the Δψm and damaging the mitochondrial structure. Mitochondria are the primary source of ROS, which are generated through the activities of the ETC in mitochondrial complexes I through IV.^[^ ^22^ ^]^ Excessive levels of ROS can cause significant mitochondrial damage, potentially leading to irreversible impairments in mitochondrial function, resulting in insufficient ATP production and oxidative damage to cellular components, triggering pro‐inflammatory responses.^[^ ^56^ ^]^ Elevated ROS levels and decreased ATP generation were observed in the hgMDV group in our study. These results suggest that MDVs can induce excessive oxidative stress under hyperglycemic conditions, leading to pathological changes. This indicates a dysfunction in mitochondrial energy metabolism, leading to impaired ATP production, one of the key mechanistic reasons behind MDV's promotion of DFU progression in the presence of hyperglycemia. In addition, SNX9‐mediated MDVs play a role in promoting inflammatory responses and are involved in the degradation of damaged mitochondrial substances under certain conditions.^[^ ^14^ ^]^ This function suggests that SNX9 may be crucial for the activity of matrix‐positive MDVs, which are associated with inflammation. Consequently, we utilized SNX9 inhibitors in our research to investigate their influence on the progression of diabetic wounds. Our studies, including animal experiments, revealed that hgMDV contributed to delayed diabetic wound healing, which could be mitigated using SNX9 inhibitors. This group of SNX9 inhibitors simulated high levels of MDV in DFU. High levels of the inhibitor were administered as a remedy, resulting in obvious effects, such as significantly accelerated wound healing, thickened granulation tissue, increased collagen deposition, and increased angiogenesis. SNX9 is most likely an important pathway for regulating the role or increased levels of MDVs in DFU. The direct assessment of glucose metabolism remains challenging in animal models. PET‐CT imaging may offer a viable approach for evaluating glucose metabolic levels in this setting. We believe that increased MDV levels in DFU are related to SNX9; thus, inhibiting SNX9 may be an important mechanism to slow the progression of DFU.

The involvement of various mechanisms in the development of chronic non‐healing wounds in patients with diabetes mellitus is well documented. Hyperglycemia induces the accumulation of intracellular ROS.^[^ ^57^ ^]^ Excessive and uncontrolled oxidative stress results in sustained and deregulated inflammation, which plays a central role in the pathogenesis of chronic non‐healing wounds.^[^ ^58^ ^]^ In diabetic wounds, ROS production through ROS‐generating enzymes is elevated, impairing wound healing processes via increased cell apoptosis and senescence with ongoing oxidative stress, lipid peroxidation, protein modification, and DNA damage.^[^ ^59^ ^]^ This oxidative stress promotes apoptosis, which is crucial in delaying wound healing.

The passage of various apoptotic and inflammatory signals via gap junctions plays an important role in tissue remodeling during diabetic wound healing. Dysregulation of apoptosis in response to hyperglycemia has been widely observed, contributing to impaired wound healing and affecting other target organs.^[^ ^60^ ^]^ During the different stages of diabetic wound healing, apoptosis is abnormal in various cell types, including fibroblasts, keratinocytes, endothelial cells, endothelial progenitor cells, neutrophils, and macrophages. Abnormal apoptosis results in uncontrolled wound inflammation, blocked angiogenesis, and impaired re‐epithelialization. Therefore, apoptosis is a key orchestrator in the remodeling of the diabetic wound healing process. Fibroblasts are heterogeneous cells that participate in the proliferation and remodeling stages of wound healing. Excessive apoptosis of fibroblasts leads to poor wound healing in hyperglycemic environments.^[^ ^61^ ^]^

Delayed chronic diabetic wound healing is intrinsically linked to sustained mitochondrial dysfunction, induced by hyperglycemia.^[^ ^62^ ^]^ Mitochondrial dynamics have been strongly associated with hyperglycemia‐induced ROS formation and vascular damage, disrupting cell integrity and barrier function, and delaying neovascularization.^[^ ^7^ ^]^ Mitochondria produce excessive mtROS and are the primary source of cellular ROS in HG environments, inducing oxidative stress and tissue damage.^[^ ^63^ ^]^ Hence, targeting mtROS production is important for improving vascular health in diabetic wounds.^[^ ^64^ ^]^ Multiple regulatory mechanisms affect mtROS production, including mitochondrial dynamics (mitochondrial fusion and fission), mitochondrial kinetics, and Δψm changes. Meanwhile, severe mitochondrial dysfunction negatively affects cell function, accelerates cell apoptosis, and increases inflammatory accumulation,^[^ ^65^ ^]^ which directly inhibits wound healing in diabetes. Our proteomic analysis corroborated these findings, revealing significant alterations in pathways related to apoptosis, metabolism, and mitochondrial function. Therefore, protecting mitochondrial function could be an innovative treatment strategy for diabetic chronic wounds.

To the best of our knowledge, there are limited reports on hgMDV in DFU. This represents the first proteomic sequencing and study of the role of MDVs in diabetic wounds. Demonstrating the similarity between tissue‐derived MDVs and those extracted from cell culture has resulted in an in vitro model to study MDV research in the context of diabetic wounds. Our work focused on the role of MDV in the progression of DFU, which has not been previously reported. Our primary starting point is that MDV is significantly elevated in DFU and, therefore, may mediate important regulatory roles. To investigate the function of MDVs, we constructed a cell model using fibroblasts; however, it is unknown exactly which cell types secrete MDVs in DFU or whether it is secreted by multiple cells. We focused only on MDVs secreted by fibroblasts and observed that MDV secretion increased under HG culture conditions, which makes it likely that MDVs mediate intracellular regulatory pathways rather than extracellular ones. We have largely elucidated the effects of MDVs on oxidative stress, glucose metabolism, and mitochondrial mass, but the specific molecules involved have not been studied. This study has some limitations. Essential MDV‐specific factors required for their formation remain unidentified, thereby limiting our understanding of their role in vivo. Further research is critical to elucidate the key regulators of MDV formation. Additionally, we failed to differentiate the composition of MDVs originating from different pathways, nor did we finely distinguish among MDVs themselves. The targets of MDVs and their specific mechanisms for regulating oxidative stress and mitochondrial dysfunction remain unexplored.

In conclusion, this study revealed a significant increase in MDVs during the transition from diabetes to DFU, a process likely regulated by *SNX9* in fibroblasts. We found that fibroblasts cultured under HG conditions could secrete MDVs with components similar to those found in the skin of DFUs, enabling the delineation of the roles of MDVs in vitro in apoptosis promotion, oxidative stress induction, mitochondrial metabolism inhibition, and mitochondrial morphology alteration. In vivo experiments revealed that SNX9 inhibitors ameliorate impaired wound healing mediated by MDVs, suggesting that MDVs are involved in DFU pathogenesis by regulating oxidative stress and mitochondrial dynamics. MDVs, which occur under hyperglycemia conditions, disrupt mitochondrial dynamics and homeostasis by reducing Δψm and damaging the mitochondrial structure. Such effects lead to mitochondrial dysfunction and, ultimately, DFU. In contrast, using an SNX9 inhibitor in a diabetic mouse model partially restores the previously described dysfunctions and promotes wound healing. Accordingly, the findings of this study highlight the underlying therapeutic effects of MDV inhibition and provide a potential target for the clinical treatment of DFU.

## 4 Experimental Section

### Purification of EVs from Human Skin Samples

The control group comprised three healthy individuals who underwent scar revision surgery, whereas the diabetes group consisted of five patients with diabetes who underwent flap grafting after accidental trauma. Five patients with DFU who underwent ulcer excision surgery were included in the DFU group. These groups provided adequate wound and peri‐wound tissues for examination. This study was approved by the Ethics Committees of Nanfang Hospital of Southern Medical University and the First Affiliated Hospital of Sun Yat‐sen University (No. [2020] 292). Information about the study participants was presented in Table S1 (Supporting Information). Clinical specimens were collected after informed consent was obtained from all participants.

Unfixed frozen skin tissue (0.5 g) from the healthy, diabetes, and DFU groups was processed for EV extraction. Frozen skin tissue was chopped on ice using a razor blade (Thermo Fisher Scientific, 12–640) to generate 2–3 mm^3^ sections, increasing the contact surface area. Next, the sections were transferred to 3 mL Earle's Balanced Salt Solution (Gibco, 14155063) containing 20 units of papain (Worthington Biochemical Corporation, LK003178), placed in a water bath at 37 °C for 15 min, and stirred once every 5 min to promote tissue breakdown. Skin tissue samples were immediately placed on ice, and 6 mL of ice‐cold DMEM was added to halt enzymatic activity and prevent overdigestion. Dissociated skin tissue samples were gently homogenized (20 strokes) using a glass Teflon homogenizer to further disrupt the tissue. The homogenized tissue samples were filtered through a 40 µm mesh filter (Coring, 352340) and centrifuged at 300 × *g* for 10 min at 4 °C to remove incompletely disrupted large tissue fragments (Eppendorf, 5720R). The supernatant was transferred to a new 15 mL tube and centrifuged at 2000 × *g* for 10 min at 4 °C (Eppendorf, 5720R). The supernatant was transferred to a 50 mL centrifugation tube and centrifuged at 10 000 × *g* for 30 min at 4 °C (Beckman Coulter, Avanti J‐E JA‐25.50). The supernatant was filtered through a 0.22 µm pore‐sized filter (Millipore Sigma, GSWP04700) to purify EVs from intracellular contaminants. The filtered liquid was placed into a 13.2 mL ultracentrifuge tube (Beckman Coulter, 331 372) and centrifuged at 100 000 × *g* for 70 min at 4 °C (Beckman Coulter, Optima XPN‐100 SW41) to precipitate EVs. After ultracentrifugation, the supernatant was discarded. The pellet contained the crude EV preparation, and the EVs were resuspended in 1 mL of phosphate‐buffered saline (PBS). The EVs obtained were used for MDV extraction.

### Detecting Protein Concentrations of EVs and MDVs

The protein concentrations of EVs (from human tissues), MDVs (from human tissues), and cultured cells (under normal and high glucose (HG) environments) were detected using a BCA protein quantitation assay kit (Beyotime, China, P0012) following the manufacturer's instructions. The same assay kit was used to measure the protein concentration of MDVs extracted from fibroblasts under various conditions. Fibroblasts were cultured under normal glucose (Fibroblast) and HG conditions (hgFibroblast). For each glucose condition, four experimental groups were established: wild type (WT), SNX9 knockdown (SNX9‐KD), SNX9 inhibition (SNX9‐IN), and SNX9 overexpression (SNX9‐OE). MDVs were isolated from each group, and their protein concentrations were quantified using the BCA assay. This experimental design allowed for the comparison of MDV protein concentrations across different glucose conditions and SNX9 expression levels.

### NTA

NTA was performed for EV and MDV particle size measurements using Particle Metrix (ZetaView Instruments, Germany) and quantified using NTA software (Particle Metrix, Meerbusch, Germany).

### Nanoscale Flow Cytometry

Nanoscale flow cytometry (nanoFCM) was used to identify the proportion of various types of vesicles by analyzing protein expression in EVs, including PEVs, EXOs/MVs, ABs, and MDV from skin tissue. NanoFCM analysis was performed using a NanoAnalyzer (NanoFCM Co. Ltd., Nottingham, United Kingdom) equipped with a 488 nm laser suitable for exciting FITC and a 647 nm laser suitable for exciting Alexa Fluor 647. For immunofluorescence staining, antibodies were applied: one part of the sample was stained with FITC Anti‐CD41 (Abcam, ab21851) and PE‐Cy7 Anti‐CD63 antibodies (Santa Cruz, sc‐5275); the other part was stained with FITC Anti‐Annexin V (Thermo Fisher, BMS147FI) and Alexa Fluor 647 anti‐translocase of outer mitochondrial membrane 20 (TOM20) antibodies (Santa Cruz, sc‐17764). Isotype control IgG was used as a negative control for the nanoFCM experiments. Each antibody was used at 1 ng µL^−1^ concentration in 50 µL of PBS. After centrifugation at 12 000 × *g* for 10 min to remove antibody aggregates, the supernatant was added to 5 × 10^5^ purified EVs and incubated for 90 min at 25 °C with constant shaking. Stained EVs were collected after centrifugation at 120 000 × *g* for 70 min and diluted 1:100 in PBS for nanoFCM analysis.

### Protein Microarray Assays

A customized protein microarray (RayBiotech Co., Ltd.) was used to detect EV biomarkers. The array was spotted with a panel of antibodies against the following proteins: apoptosis‐linked gene 2‐interacting protein X (ALIX), PDH, TOM20, heat shock protein 70, P‐selectin, CD41, annexin V, CD63, and CD9. EV samples were lysed in RIPA buffer supplemented with protease inhibitor cocktail on ice to fully disrupt vesicle membranes and solubilize proteins for subsequent analysis. The lysates were centrifuged at 15 000 × *g* for 20 min at 4 °C, and the supernatant containing soluble proteins was collected. The protein concentration was determined using a BCA protein assay kit (Beyotime, P0012). All samples were normalized to equal volumes and concentrations, and 2 µg of protein was loaded per well to ensure consistency in subsequent applications. The array membranes were blocked with 500 µL blocking buffer at room temperature for 1 h with gentle shaking to avoid bubble formation. After removing the blocking buffer, samples were added to each well and incubated overnight at 4 °C with gentle agitation. After the EVs were bound to the primary antibodies on the custom protein microarray, the microarray was washed with 500 µL washing buffer at room temperature to remove non‐specifically bound EVs. The microarrays were then incubated with HRP‐conjugated antibodies for 2 h at room temperature for colorimetric detection, followed by repeated washing steps. For detection, microarrays were developed using ECL chemiluminescent substrate with 2 min incubation, and signals were captured using an Azure Biosystems imaging system. The microarray was developed using a chromogenic substrate, and the colorimetric signal was measured to determine the presence and abundance of target EV proteins. The expression levels of biomarker proteins were compared with the gray values of 1 µg mL^−1^ HRP spots using relevant spot gray values. Quantitative gray values of the chromogenic substrates were analyzed using ImageJ software (NIH, USA). DEPs were represented as heat maps generated using GraphPad Prism software.

### Western Blot Analysis

EVs from human and mouse tissues and cultured cells were collected for protein extraction. Total proteins were extracted using RIPA lysis buffer (Solarbio, China, R0010) supplemented with a protease inhibitor cocktail (Beyotime, P1005). EVs isolated from human skin samples were resuspended in 100 µL PBS and mixed with 300 µL RIPA buffer. Approximately 50 mg of mouse skin wound tissue was homogenized in 500 µL RIPA buffer using a tissue grinder. For the treated cells, the culture medium was removed. Cells were washed twice with ice‐cold PBS, and then lysed directly in a culture dish with RIPA buffer (100 µL/10^6^ cells). All samples were incubated on ice for 30 min with occasional vortexing and then centrifuged at 14 000 × *g* for 15 min at 4 °C. Supernatants containing total proteins were collected and quantified using a BCA kit (Beyotime, P0012) according to the manufacturer's instructions. Protein lysates were separated using 10% SDS‐PAGE and transferred onto a polyvinylidene fluoride membrane (Merck Millipore, IPVH00010). The transferred membranes were blocked using a 5% skim milk solution for 1 h. The membranes were then incubated overnight at 4 °C with specific primary antibodies against various targets. Detailed information on the antibodies used in this study was presented in Table S2 (Supporting Information). The next day, the membranes were washed with TBST solution and incubated with the corresponding secondary antibody (Merck Millipore, AP132P) for 1 h at 25 °C. Finally, enhanced chemiluminescence reagents (Beyotime, P0018S) were used for visualization, and ImageJ software was used for quantitative analysis.

### Acquisition and Processing of Data from a Single Cell

Data from the publicly available DFU single‐cell mRNA sequencing dataset, GSE165816, were obtained from a research group led by Georgios Theocharidis. This study analyzed processed transcriptomic data from 24 ft skin samples, including those from 11 healthy individuals, 8 patients with diabetes without DFU, and 5 with non‐healing DFU, as the healthy, diabetes, and DFU groups, respectively. Sequencing of single cells from these samples yielded 66365 cells that were retained after data processing procedures, including quality control, data filtering, and normalization. The sequencing was performed using the Illumina HiSeq 4000 platform (Illumina, California, USA). UMAP analysis was set to a resolution of 0.9, and the “FindClusters” function was employed to classify the cells into 11 distinct clusters. We investigated the expression of genes involved in the biogenesis of MDVs, including RAB9A, PRKN, VPS35, STX17, TOLLIP, DNM1L, SNX9, and OPA1 across these clusters and within the healthy, diabetes, and DFU groups.

### Quantitative PCR (qPCR) Analysis of Skin Tissue Gene Expressions

Quantification was performed for RAB9A, PRKN, VPS35, STX17, TOLLIP, DNM1L, SNX9, and OPA1. Before RNA isolation, all skin tissue samples were trimmed into smaller pieces using scissors. Total RNA was extracted from skin tissue lysates using an RNAiso Plus kit (Takara, Japan, 9108Q). For qPCR analysis, the SYBR Green Real‐Time PCR Master Mix (Thermo Fisher Scientific, 4309155) and specific primers supplied by Beyotime were used. Gene expressions were calculated using the formula 2^−△△Ct^. The primer sequences were listed in Table S3 (Supporting Information).

### Immunoadsorption Separation of MDVs

The magnetic beads (absin, abs9905) were prewashed with PBS to remove the storage buffer and reduce nonspecific binding. A magnetic stand was used to separate the beads from the PBS. The supernatant was discarded, and the washing step was repeated twice. The prewashed magnetic beads were incubated with an anti‐TOM20 antibody (Santa Cruz, sc‐17764) using 5 µg per mg of beads with gentle rotation for 1 h at room temperature to allow antibodies to bind to the beads. After the antibody binding, the beads were washed three times with IP buffer to remove any unbound antibodies. Previously isolated EVs (EVs from skin tissues or cell culture medium, 1 mg mL^−1^) were added to the antibody‐conjugated beads. The EV concentrations were determined using the BCA method and diluted to ensure consistency. The mixture was incubated with gentle rotation overnight at 4 °C to allow the TOM20 antibodies to bind to MDVs. After incubation, the tube was placed on a magnetic stand to collect the beads bound to MDVs, and the supernatant containing unbound EVs was removed. Bead‐bound MDVs were washed three times with an ice‐cold IP buffer to remove unbound EVs. After the final wash, PBS was added to the beads and gently resuspended to collect bead‐bound MDVs for downstream analyses, such as electron microscopy or proteomic analysis. If intended for cell culture, serum‐free DMEM was used for resuspension. The isolated MDVs were stored at −80 °C for long‐term storage.

### Primary Fibroblast Isolation, Culture, and Treatment

Primary fibroblasts were isolated from the skin of neonatal C57BL/6 mice^[^ ^33^ ^]^ and cultured in a complete medium containing 10% exosome‐free fetal bovine serum (FBS) (Gibco, USA, A2720803) and 1% penicillin/streptomycin (Gibco, 15140122) at 37 °C with 5% CO_2_. The cells were used for the experiments upon reaching 80%–90% confluence in appropriate culture dishes. All tests were conducted on cells between passages three and five. Fibroblasts were cultured in HG medium containing 35 mM d‐(+)‐glucose for 48 h to assess the effects of HG. Conversely, the control group received a complete medium containing 5.5 mM glucose. For the HG environment, we initially prepared an HG medium by diluting a D‐glucose solution (200 g L^−1^) from Pricella (PB180418) with a serum‐free medium to achieve a glucose concentration of 35 mM. The medium was then supplemented with exosome‐free FBS to adjust the serum concentration to 10%. The cells were cultured in an HG medium for 48 h to assess the impact of HG on fibroblast behavior. This setup enabled us to directly compare cellular responses under physiological and HG conditions, facilitating an accurate evaluation of the metabolic effects induced by hyperglycemia. The supernatant was collected after the cell culture reached an appropriate density.

The experiment consisted of three groups: Control, MDV and hgMDV. In all groups, HG fibroblasts were cultured in T25 flasks containing 4 mL of complete medium. In the Control group, HG fibroblasts were cultured without additional treatment. For the MDV and hgMDV groups, HG fibroblasts were initially seeded in T25 flasks containing a complete medium. Subsequently, the treatment solutions were added to the respective flasks: MDV solution for the MDV group and hgMDV solution for the hgMDV group. The final concentration of MDVs or hgMDVs in the culture medium was 60 µg mL^−1^ for both treated groups. To ensure consistent exposure to MDVs, the culture medium was replaced daily with a fresh medium containing the same concentration of MDVs (60 µg mL^−1^).

Mycoplasma testing was consistently conducted throughout the experimental process as part of routine cell quality control, with weekly tests. Cells were specifically cultured in 75 cm^2^ tissue culture flasks, and mycoplasma testing was carried out using the Myco‐Lumi Luminescent Mycoplasma Detection Kit (Beyotime, C0298S), following the manufacturer's protocol.

### TEM

TEM was used to analyze MDV morphology. For 1 min, 10 µL of purified MDVs was added to a copper grid. The samples were negatively stained with 2% uranyl acetate for 1 min and dried at room temperature. MDV images were captured using a TEM at 80 kV (HT7700, HITACHI, Japan). To observe the mitochondrial morphology, cells were carefully washed and immersion‐fixed with a 2.5% glutaraldehyde solution for 8 h at 4 °C. Subsequently, the fixation solution was removed, and cells were incubated with 1% osmium tetroxide for 2 h at 4 °C. The cells were dehydrated using an acetone gradient and soaked in araldite resin. Ultrathin sections were prepared and counterstained using uranyl acetate and lead citrate. Mitochondrial ultrastructural analysis was performed using TEM.

### Proteomics Analysis

Furthermore, label‐free quantitative proteomic analysis was conducted to ascertain the composition of the MDVs using services provided by LC Sciences (Hangzhou, Zhejiang, China). This process involved protein extraction and trypsin digestion, after which the peptides were dissolved in liquid chromatography mobile phase A. The peptides were separated using a NanoElute ultra‐performance liquid chromatography system and injected into the capillary ion source for ionization, followed by TIMS‐TOF Pro mass spectrometry analysis. Using heatmap analysis, we compared the protein content of MDVs from healthy individuals, patients with diabetes, and MDVs derived from cells under normal and HG conditions. Correlation analyses were performed to evaluate the interrelationships among samples. Differential analyses were performed on the DFU versus healthy groups and the hgMDV versus MDV groups, and the differential protein sets’ intersection was illustrated using a Venn diagram. GO analysis was employed to assess the functional properties of the different proteins, and KEGG pathway enrichment analysis was conducted to elucidate these proteins’ roles in cellular processes. Statistical significance was set at *p* < 0.05.

### Uptake of MDVs

100 µg MDVs were labeled with 5 µL EvLINK 505 (TINGO, EL012100200) following the manufacturer's protocol and incubated with gentle mixing at room temperature in the dark for 30 min. To remove excess dye, the green fluorescently labeled MDVs were ultrafiltered at 12 000 × *g* for 15 min using a 100 kDa ultrafiltration unit (Millipore, UFC910096). The purified sample was collected for subsequent experiments to assess MDV uptake by fibroblasts. Fibroblasts were seeded into confocal dishes and cultured for 24 h. Subsequently, fibroblasts were incubated with EvLINK 505‐labeled MDVs for 12, 24, 36, and 48 h and washed with PBS. To visualize the cell membrane, fibroblasts were labeled with CellLINK 555 (TINGO, CL012100221) and incubated at room temperature in the dark for 30 min. Following incubation, cells were washed with PBS and fixed with 4% paraformaldehyde for 30 min. The nuclei were then stained with 4′,6‐diamidino‐2‐phenylindole (DAPI, Solarbio, C0060) for 5 min. The uptake of labeled MDVs was observed using a confocal laser scanning microscope (LSM980, Zeiss). The acquired images were analyzed using ImageJ software to quantify the relative fluorescence intensities.

### TUNEL Assay

Fibroblasts were analyzed for apoptosis using a TUNEL fluorescence detection kit (Beyotime, C1086). Fibroblasts on glass bottom dishes were fixed with 4% paraformaldehyde for 15 min, permeabilized with 0.5% Triton X‐100 for 20 min, and blocked with goat serum for 30 min at 37 °C. After washing three times with PBS, 50 µL of TUNEL test solution was added to each well. Incubation continued at 37 °C for 1 h in the dark, followed by three PBS washes. DAPI was applied to cells for 10 min at room temperature. Stained cells were examined using a confocal laser scanning microscope.

### Cellular ROS Detection

ROS levels were measured in fibroblasts treated with MDVs and hgMDVs for 3 days using a ROS testing kit (Beyotime, S0033S). The fibroblasts were seeded in glass‐bottomed dishes according to the manufacturer's instructions. Each group of cells was incubated with a 10 µM concentration of the 2′,7′‐dichlorodihydrofluorescein diacetate (DCFH‐DA) probe for 30 min. The cells were then washed three times with PBS and observed under a confocal laser microscope. The mean fluorescence intensity of the images was quantified using the ImageJ software (Media Cybernetics Inc., USA).

### Biochemical Assays of Oxidative Stress

Intracellular MDA levels were assessed using a cellular lipid peroxidation MDA assay kit (Nanjing Jiancheng Bioengineering Institute, China, A003‐1‐2). Intracellular SOD and CAT activities were measured using SOD activity assay kits (Solarbio, BC0170) and CAT activity assay kits (Solarbio, BC0200), respectively. All procedures were performed following the manufacturer's instructions.

### Detection of Mitochondrial Membrane Potential

To assess Δψm in the Control, MDV, and hgMDV groups cultured for 3 days, a JC‐1 mitochondrial membrane potential kit (Solarbio, M8650) was used. Cells from each group were seeded in confocal dishes. JC‐1 staining solution was added following the manufacturer's instructions, and the cells were incubated at 37 °C for 15 min. Confocal laser microscopy was used to capture images of JC‐1 monomers and aggregates at 488 and 594 nm, respectively. The ratio of red to green fluorescence intensity was calculated to quantify the fluorescence images corresponding to the polymeric and monomeric forms.

### Mito‐Tracker Staining

The cells in each group were cultured overnight in confocal dishes. After treatment, cells were stained with a 200 nM MitoTracker Red solution (Solarbio, M9940) and incubated for 20 min at 37 °C in 5% CO_2_. Images were obtained using a laser confocal microscope at 594 nm and analyzed using ImageJ software.

### Detection of Glucometabolic Levels

After 3 days of culture, the metabolites of each group were quantified using specific kits: ATP levels with an ATP assay kit (Beyotime, S0026), lactic acid concentrations with lactate assay kit (Dojindo, L256), and pyruvic acid levels with pyruvic acid colorimetric assay kit (Elabscience, E‐BC‐K130‐M). The activities of glycolysis‐related enzymes, namely, PFK‐1 (Saint‐bio, BA1194), HK (Saint‐bio, BA2147), PK (Saint‐bio, BA1061), LDH (Saint‐bio, BA1260), and PDH (Saint‐bio, BA1059), were determined using their respective specific activity detection kits. The mRNA expression levels of enzymes involved in the TCA cycle, including α‐KGDH, IDH, and CS, were quantified using RT‐qPCR. RT‐qPCR was used to measure the mRNA levels of key components of the mitochondrial ETC: NDUFB3, MTCO3, and SDHB. The qPCR reaction mixture included 5 µL SsoFast Eva Green Supermix (Bio‐Rad, #1 725 200), 0.5 µL of each 10 µM primer, 2 µL cDNA template, and 2 µL of ultrapure water. GAPDH was used as the reference gene for normalization. Relative gene expression was determined using the 2^−∆∆CT^ method. The primer sequences used were listed in Table S3 (Supporting Information).

### Knockdown, Overexpression, and Inhibitor Intervention of SNX9

Fibroblasts were cultured under two conditions: normal glucose (5.5 mM, referred to as the fibroblast group) and HG conditions (35 mM, referred to as the hgfibroblast group), and the effects of SNX9 knockdown and overexpression were evaluated. In this setup, untreated cells served as the WT group (no intervention), while the SNX9 knockdown group was referred to as the SNX9‐KD group, and the SNX9 overexpression group was referred to as the SNX9‐OE group. Additionally, a group treated with an SNX9 inhibitor at a concentration of 5 µM was included, referred to as the SNX9‐IN group. The SNX9‐IN group was cultured in a medium supplemented with 5 µM of the SNX9 inhibitor for 24 h before analysis. This experimental design allowed us to compare the impact of SNX9 modulation under both normal and HG conditions and the effect of pharmacological inhibition of SNX9.

To knock down SNX9 expression, a specific siRNA targeting SNX9 (#45 090 174) was purchased from Applied Biological Materials Inc. Fibroblasts were seeded in six‐well plates and allowed to adhere for 24 h. For transfection, Lipofectamine 2000 (Thermo Fisher Scientific, 11 668 030), a cationic lipid reagent, was used according to the manufacturer's instructions. Fibroblasts were transfected with 50 nM siRNA for 24 h. After transfection, the cells were harvested for protein extraction and subsequently used for western blot analysis to assess SNX9 expression levels.

The full‐length coding sequence of the SNX9 gene (NM_025664) was amplified using PCR. After amplification, PCR products were purified and subjected to restriction enzyme digestion. The digested product was purified and ligated into the linearized pcDNA3.1 vector. After ligation, the plasmid was transformed into *E. coli*, and the bacteria were plated onto agar plates containing ampicillin and incubated at 37 °C for 15 h. After incubation, individual colonies were selected and screened using colony PCR to verify the presence of the insert. Positive clones were sequenced to confirm the accuracy of the insert sequence. To overexpress SNX9, fibroblasts were seeded into six‐well plates at an appropriate density 24 h before transfection, ensuring that they reached 90%–95% confluence at the time of transfection. Transfection was performed using 4 µg of pcDNA3.1‐SNX9 plasmid DNA and Lipofectamine 2000 as the transfection reagent. After 6 h of incubation, the transfection medium was replaced with fresh complete medium containing serum. The cells were cultured for 24 h at 37 °C in a 5% CO_2_ atmosphere. Fibroblasts were harvested and subjected to western blot analysis to assess SNX9 expression levels. Following these experimental manipulations (SNX9 knockdown, overexpression, and inhibition), MDVs were extracted from all experimental groups according to the previously described method. The isolated MDVs were analyzed to characterize their properties. NTA was performed to determine the size distribution and concentration of the extracted MDVs, and TEM was employed to visualize the morphology of the isolated MDVs. Additionally, the protein concentration of the extracted MDVs was quantified using a BCA protein assay kit, following the manufacturer's instructions.

### In Vivo Model of Diabetic Wound Healing

Animal care and treatment were conducted following institutional procedures and national laws and regulations. The animal experimental protocol, approved by Nanfang Hospital, Southern Medical University (IACUC‐LAC‐20240111‐002), was strictly followed for all animal feeding and experimental procedures following the requirements of the Animal Ethics Committee. To induce type 1 diabetes, male C57BL/6 mice (6–8 weeks, 16–20 g) were intraperitoneally injected with streptozotocin (STZ, Macklin, S817944) at a dose of 50 mg kg^−1^ for five consecutive days after fasting overnight. Two weeks after the last STZ injection, fasting blood glucose levels were measured using a glucometer (GA‐3; SinoCare). Mice with fasting blood glucose levels exceeding 16.7 mM were classified as diabetic. Mice were anesthetized, and their dorsal hair was shaved. A 10 mm‐diameter, full‐thickness cutaneous wound was created on each mouse using a punch biopsy.

Thirty‐six C57BL/6 mice were randomly divided into four groups: Control, Diabetes, hgMDV, and hgMDV + SNX9‐IN. Three mice from each group were randomly euthanized at each of the predetermined time points (days 3, 7 and 14). The control group did not receive STZ and was left untreated; the diabetes group received saline on wounds; the hgMDV group received 25 µL of hgMDV solution at a concentration of 60 µg mL^−1^ applied to the wounds; the hgMDV+SNX9‐IN group received 25 µL of 60 µg/mL hgMDV treatment and an SNX9 inhibitor (DATPT, HY‐145307, MedChemExpress) at a concentration of 800 µg mL^−1^, administered at a volume of 1.25 µL per gram of body weight to wounds following confirmation of diabetes. The treatment for each group was administered by directly dropping it onto the wound area, beginning on the first day following wound creation and continuing daily until day 7. At predetermined time points, the mice were humanely euthanized, and the wound tissues were collected for analysis. Concurrently, the wound area was photographed, and the size was quantified using ImageJ software.

### Histological Evaluation

Wound tissues from different experimental groups were fixed with 4% paraformaldehyde, embedded in paraffin, and cross‐sectioned into 4 µm‐thick slices. Sections were mounted on glass slides and baked at 60 °C for 2 h and stained with hematoxylin and eosin (H&E) (Beyotime, C0105S) and Masson's trichrome (Solarbio, G1340) for histological analysis, following the manufacturer's standard protocols. After staining, all sections were dehydrated, cleared, and mounted for microscopic examination. ImageJ software was used to quantitatively analyze the wound healing parameters, including the length of the wound area, epidermal thickness, granulation tissue thickness, and collagen deposition.

For immunohistochemical (IHC) analysis, sections were deparaffinized in xylene and rehydrated using a graded ethanol series. Antigen retrieval was performed using citrate buffer (pH 6.0) in a pressure cooker for 10 min. Endogenous peroxidase activity was quenched with 3% H_2_O_2_ for 10 min. The sections were blocked with 5% goat serum for 20 min and incubated with primary antibodies at a dilution of 1:200 overnight at 4 °C. The antibodies used for IHC were anti‐Collagen I (Proteintech, 14695‐1‐AP), anti‐Collagen III (Proteintech, 22734‐1‐AP), SOD1 (Proteintech, 10269‐1‐AP), and cleaved caspase‐3 (Proteintech, 25128‐1‐AP). Following washing, sections were incubated with HRP‐conjugated secondary antibodies (ZSGB‐BIO, PV‐9000). Color development was conducted using a DAB substrate kit (ZSGB‐BIO, ZLI‐9018). The sections were counterstained with H&E, dehydrated, cleared, and mounted with neutral balsam. For immunofluorescence staining, a protocol similar to that used for IHC was followed, but with fluorophore‐conjugated secondary antibodies. NRF1 (Abcam, ab34682, 1:200) and PINK1 (Abcam, ab216144, 1:200) were detected using goat anti‐rabbit IgG H&L (Alexa Fluor 488) secondary antibody (Abcam, ab150077) at a dilution of 1:500 and incubated for 20 min at room temperature. IHC slides were imaged using a light microscope (model to be specified), while immunofluorescence slides were visualized using a laser confocal microscope (LSM980). The staining intensity and distribution were quantified using ImageJ software. Each experiment was repeated at least three times, and statistical analysis was conducted using one‐way ANOVA.

### Proteomics and Data‐Independent Acquisition‐Based Quantification

To investigate the mechanisms by which hgMDV and SNX9 affect wound healing, classical bottom‐up proteomics combined with data‐independent acquisition of fragment spectra for label‐free quantification was performed. Skin wound samples were collected from three groups of mice (Diabetes, hgMDV, and hgMDV+SNX9‐IN), with three replicates per group. After pretreatment, the samples were transferred to 1.5 mL tubes, followed by adding lysis buffer (1% SDC, 1% protease inhibitor). Samples were sonicated and centrifuged at 12 000 × *g* for 10 min at 4 °C. The supernatants were then transferred to fresh tubes, and the protein concentrations were determined using a BCA assay. Equal amounts of protein from each sample were digested with trypsin, following reduction with DTT and alkylation with IAA. Digestion was performed overnight, followed by a second digestion step for 4 h. Peptides were dissolved in mobile phase A (0.1% formic acid and 2% acetonitrile) and separated using a NanoElute UHPLC system. A gradient of 6%–80% mobile phase B (0.1% formic acid in acetonitrile) was applied for 15 min at a flow rate of 500 nL min^−1^. The separated peptides were ionized using a capillary ion source and analyzed using a TIMSTOF Pro 2 mass spectrometer. Data were acquired in the dia‐PASEF mode with an MS scan range of 300–1500 m z we performed, followed by 20 PASEF scans for fragment ions (400‐850 m z^−1^). Proteomic analysis was conducted by Jingjie PTM BioLab (Hangzhou).

### Statistical Analysis

Data from at least three independent experiments are presented as the mean values and corresponding standard deviation. Statistical comparisons between groups were performed using either an independent Student's *t*‐test for two groups or one‐way ANOVA for more than two groups based on the specific analysis required. All statistical analyses were performed using GraphPad Prism (version 10.0). Differences between groups were considered statistically significant at *p < 0.05, **p < 0.01, ***p < 0.001, and ****p < 0.0001; “ns” indicated not significant.

## Conflict of Interest

The authors declare no conflict of interest.

## Author Contributions

H.Z., Z.Y., and J.Z. contributed equally to this work. S.L., W.H., and L.Y. performed conceptualization; H.Z., Z.Y., J.Z., and Z.L. performed data curation and formal analysis; H.Z., L.C., W.Z., Z.D., S.L., W.H., and L.Y. performed methodology; H.Z., J.Y., X.Y., and Y.W. performed investigation; H.Z., H.Z., Z.J., and Q.Y. performed visualization; S.L., W.H., and L.Y. performed supervision; H.Z., Z.Y., J.Z., and Z.L. wrote the original draft; H.Z., S.L., W.H., and L.Y. wrote, reviewed and edited.

## Supporting information



Supporting Information

## Data Availability

The data that support the findings of this study are available from the corresponding author upon reasonable request.
