# Supplementary material for: Extracellular Mitochondrial‐Derived Vesicles Affect the Progression of Diabetic Foot Ulcer by Regulating Oxidative Stress and Mitochondrial Dysfunction
Source: Adv Sci (Weinh). 2025 Jan 21;12(10):2407574. doi: 10.1002/advs.202407574 (PMC11904950; doi:10.1002/advs.202407574)
Supplement: Supplementary file 1 — Supporting Information [file ADVS-12-2407574-s001.docx]

Supporting Information

**Extracellular** **Mitochondrial-derived Vesicles Affect** **Diabetic Foot Ulcer** **Progression by Regulating** **Oxidative Stress and** **Mitochondrial Dysfunction**

**Huihui Zhang****^1,^****^2†^, Zi Yan^2,3,4†^, Junyou Zhu****^5†^, Ziyue Li^2,4^, Lianglong Chen^1^, Weihan Zheng^2,4^, Zhenning Dai^6^, Jiaxin Yang^2^, Xinyi Yun^2^, Yilin Wang^2^, Hai Zhou****^1^, Ziwei Jiang^1^, Qiuyi Yu^1^, Shiyu Li^3*^, Wenhua Huang****^2, 4*^, Lei Yang^1*^**

**Affiliation**

^1^Department of Burns, Nanfang Hospital, Southern Medical University, Guangzhou, 510515, China.

^2^Guangdong Engineering Research Center for Translation of Medical 3D Printing Application, Guangdong Provincial Key Laboratory of Digital Medicine and Biomechanics, National Key Discipline of Human Anatomy, School of Basic Medical Sciences, Southern Medical University, Guangzhou, China.

^3^Department of Microbiology and Immunology, College of Basic Medicine and Public Hygiene, Jinan University, Guangzhou, 510632, China.

^4^Guangdong Medical Innovation Platform for Translation of 3D Printing Application, The Third Affiliated Hospital of Southern Medical University, Southern Medical University, Guangzhou, 510630, China.

^5^Department of Burns, First affiliated hospital, Sun Yat-sen University, Guangzhou 510080, China.

^6^Department of Stomatology, Guangdong Provincial Key Laboratory of Research and Development in Traditional Chinese Medicine, Guangdong Second Traditional Chinese Medicine Hospital, Guangzhou 510095, China.

***Corresponding authors: Shiyu Li, E-mail: lishiyu@jnu.edu.cn; Wenhua Huang, E-mail: huangwenhua2009@139.com; Lei Yang, E-mail: yuanyang@smu.edu.cn.**

†These authors have contributed equally to this work.

**
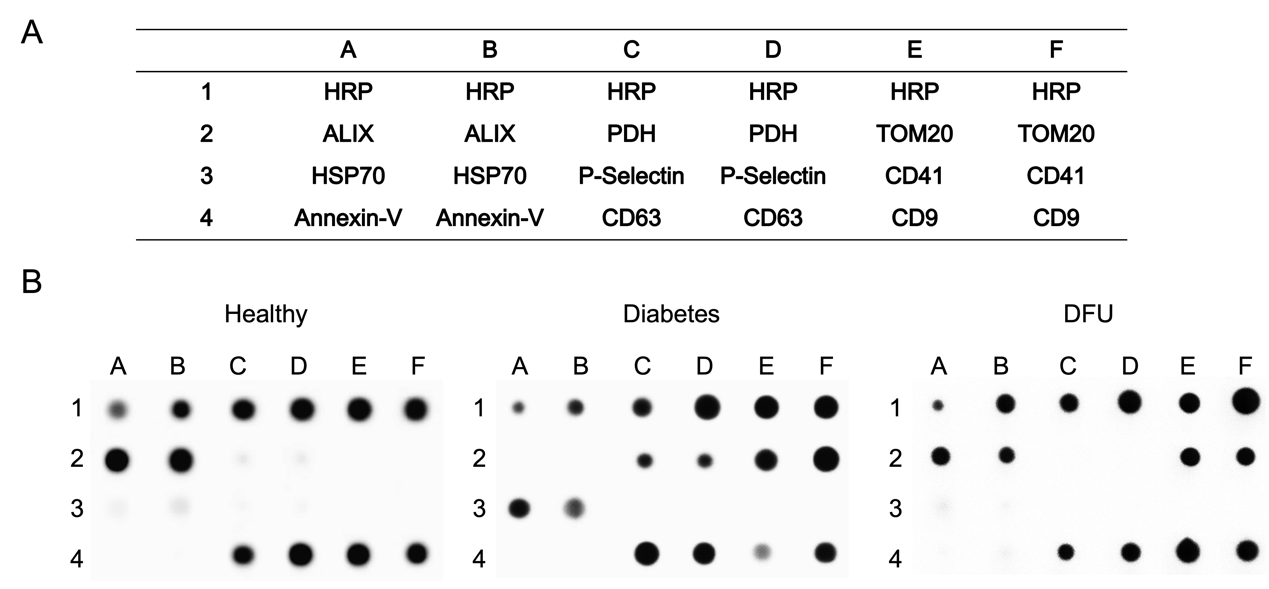
**

**Figure S1.** Protein microarray analysis of skin tissue EVs. (A) Template illustrating the protein location on a protein microarray membrane. (B) Protein microarray of skin tissue EVs.

**
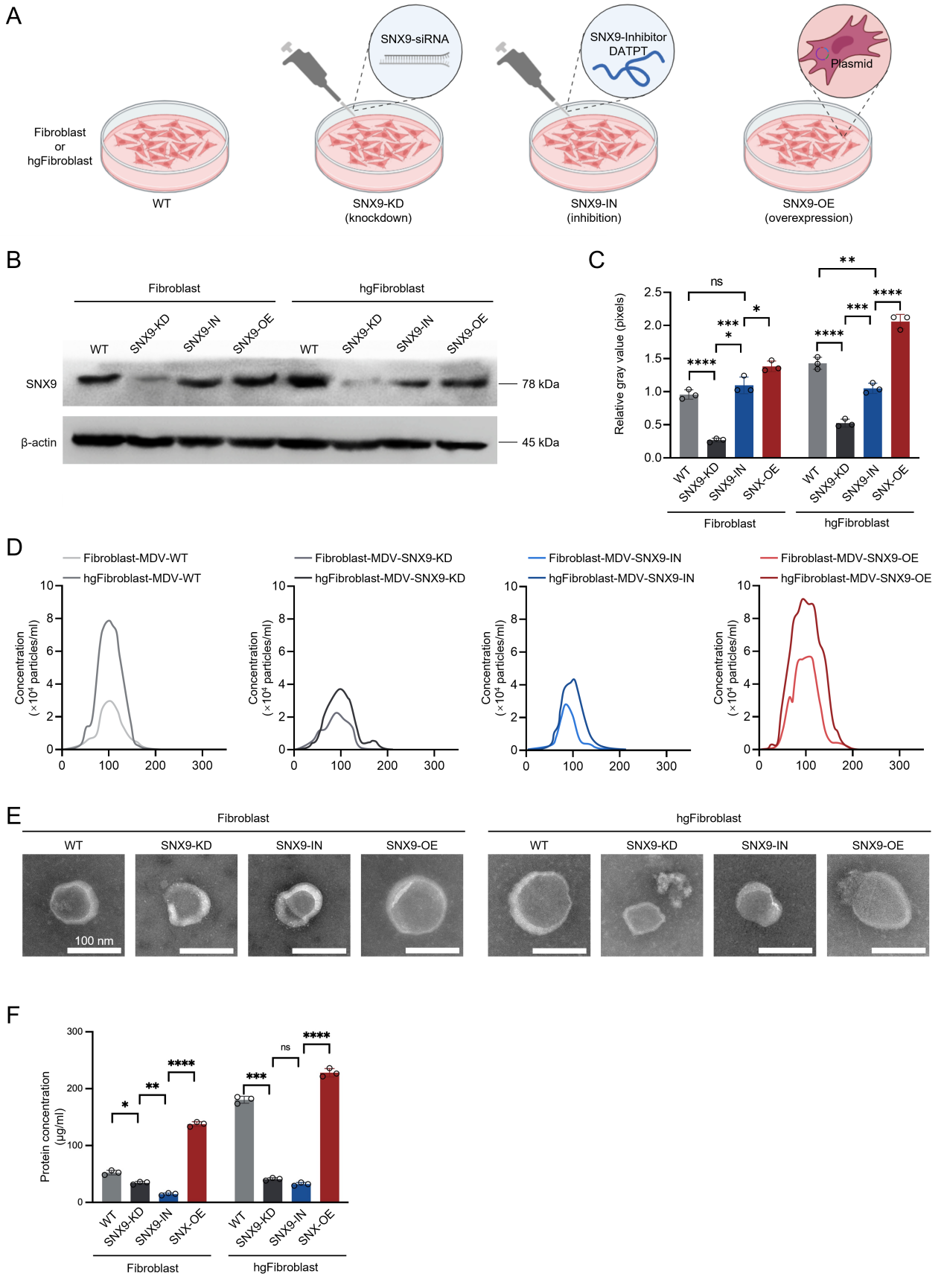
**

**Figure S2.** The impact of SNX9 on MDV production. (A) Schematic representation of experimental conditions: wild-type (WT), SNX9 knockdown (SNX9-KD), SNX9 inhibition (SNX9-IN), and SNX9 overexpression (SNX9-OE) in Fibroblasts or hgFibroblasts. (B) Western blot analysis of SNX9 protein levels. (C) Quantification of relative SNX9 protein levels from Western blot analysis. ns: *p*>0.05; n=3. Data are presented as the mean ± SD. *: *p*<0.05; **: *p*<0.01; ***: *p*<0.001; ****: *p*<0.0001. (D) NTA showing concentration of MDV produced by Fibroblasts and hgFibroblasts under different SNX9 modulation conditions. (E) Representative TEM images of MDV isolated from Fibroblasts and hgFibroblasts under different experimental conditions. Scale bars: 100 nm. (F) The protein level of MDVs was evaluated using a BCA protein assay kit.

**
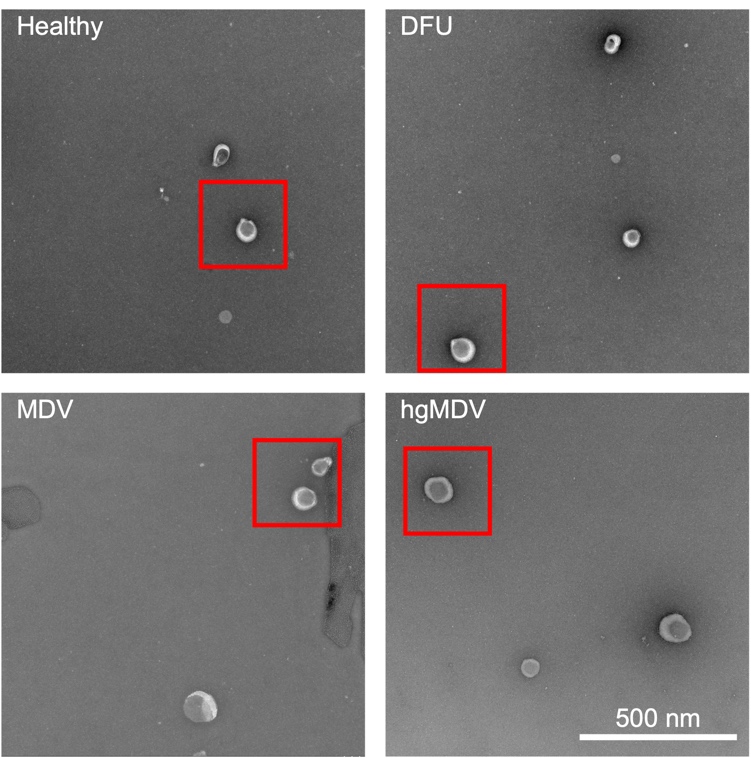
**

**Figure S3.** Wide-field view of MDVs captured by TEM. This image corresponds to the smaller field shown in Figure 3A. The highlighted red boxes indicate the magnified areas seen in Figure 3A. This provides a broader context of the MDV population in healthy, DFU, normal glucose (MDV), and high glucose (hgMDV) conditions. Scale bar: 500 nm.

**
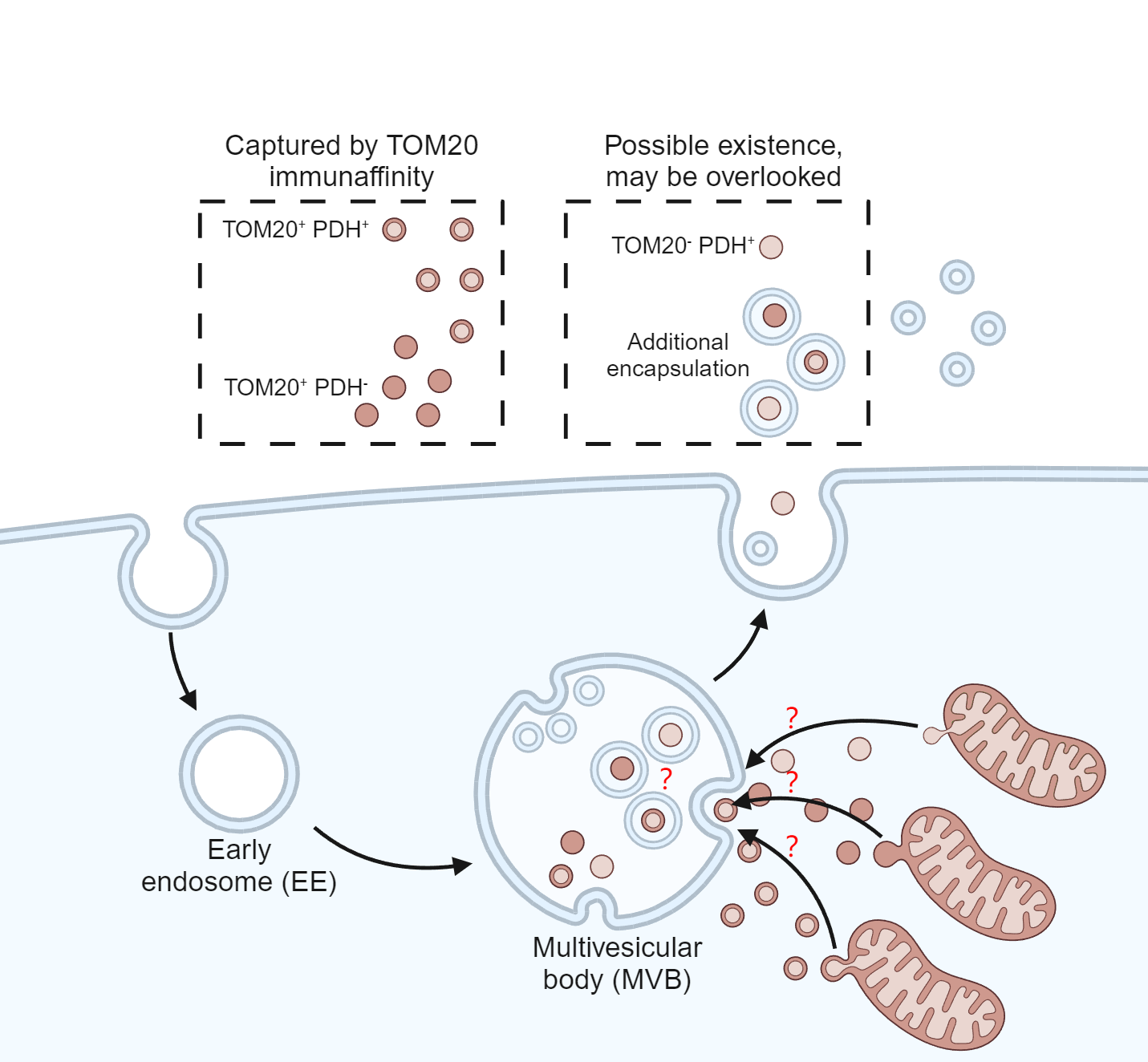
**

**Figure S4:** Schematic representation of the interactions and possible fates of TOM20^+^ and TOM20⁻ vesicles within cells. The vesicles captured by TOM20 immunoaffinity are categorized as TOM20^+^ PDH^+^ and TOM20^+^ PDH⁻. Additionally, if there are additional encapsulated MDVs or TOM20⁻ MDVs in the extracellular space, the TOM20 immunoaffinity separation method used in this study may miss these MDVs.

**Table S1**: Information of Study Participants

| Subject | Gender | Age | Population |
| --- | --- | --- | --- |
| Healthy1 | Male | 50 | Asian |
| Healthy2 | Female | 48 | Asian |
| Healthy3 | Male | 42 | Asian |
| Diabetes1 | Male | 66 | Asian |
| Diabetes2 | Male | 53 | Asian |
| Diabetes3 | Female | 68 | Asian |
| Diabetes4 | Male | 68 | Asian |
| Diabetes5 | Male | 57 | Asian |
| DFU1 | Female | 63 | Asian |
| DFU2 | Male | 58 | Asian |
| DFU3 | Male | 60 | Asian |
| DFU4 | Male | 62 | Asian |
| DFU5 | Female | 55 | Asian |

**Table S2**: Antibodies

| Antibodies | Company | Dillution | Catalogue Number |  |
| --- | --- | --- | --- | --- |
| FITC Anti-CD41 | Abcam | 1 ng/μL | ab21851 |  |
| PE-Cy7 Anti-CD63 | Santa Cruz | 1 ng/μL | sc-5275 |  |
| FITC Anti-Annexin V | Thermo Fisher | 1 ng/μL | BMS147FI |  |
| Alexa Fluor 647 Anti- TOM20 | Santa Cruz | 1 ng/μL | sc-17764 |  |
| Anti-Collagen I | Proteintech | 1:200 for IHC | 14695-1-AP |  |
| Anti-Collagen III | Proteintech | 1:200 for IHC | 22734-1-AP |  |
| Anti-CD31 | Proteintech | 1:200 for IF | 28083-1-AP |  |
| NRF1 | Proteintech | 1:500 for WB  1:200 for IF | 12482-1-AP |  |
| PINK1 | Proteintech | 1:500 for WB  1:200 for IF | 23274-1-AP |  |
| SOD1 | Proteintech | 1:5000 for WB  1:200 for IHC | 10269-1-AP |  |
| Caspase-3 | Proteintech | 1:500 for WB | 19677-1-AP |  |
| Cleaved Caspase-3 | Proteintech | 1:500 for WB  1:200 for IHC | 25128-1-AP |  |
| CAT | Proteintech | 1:2000 for WB | 21260-1-AP |  |
| NRF1 | Proteintech | 1:500 for WB | 12482-1-AP | |
| β-actin | Proteintech | 1:4000 for WB | 20536-1-AP |  |
| SNX9 | Proteintech | 1:1000 for WB | 15721-1-AP |  |
| CD9 | ABclonal | 1:2000 for WB | A1703 |  |
| CD63 | ABclonal | 1:200 for WB | A5271 |  |
| CD81 | Signalway Antibody | 1:500 for WB | 41779 |  |
| TOM20 | Proteintech | 1:5000 for WB | 11802-1-AP |  |
| CD42a | Proteintech | 1:2000 for WB | 14564-1-AP |  |
| C1QC | Proteintech | 1:2000 for WB | 16889-1-AP |  |
| IgG | Proteintech | 1:2000 for WB | 10284-1-AP |  |
| Albumin | Proteintech | 1:2000 for WB | 16475-1-AP |  |
| Goat anti-Rabbit IgG Peroxidase Conjugated | Merck Millipore | 1:2000 for WB | AP132P |  |
| Goat Anti-Rabbit IgG H&L (Alexa Fluor® 488) | abcam | 1:500 for IF | ab150077 |  |

IF: Immunofluorescence; IHC: Immunohistochemistry; WB: Western Blot

**Table S3.** Primer sequences used for qRT-PCR

| Gene | Primer Sequences (F = forward; R = reverse) |
| --- | --- |
| RAB9A | F: 5′-AGGGACAACGGCGACTATC-3′ |
|  | R: 5′-TCTGACCTATCCTCGGTAGCA -3′ |
| PRKN | F: 5'-TCCTCCACTGTTTCACGATGG-3' |
|  | R: 5'-TTGAGGCGGAAGTCTTGCAC-3' |
| VPS35 | F: 5'-GTCAAGTCATTTCCTCAGTCCAG-3' |
|  | R: 5'-CCCCTCAAGGGATGTTGCAC-3' |
| STX17 | F: 5'-GTGAAATTACGCCGTCTTGAAC-3' |
|  | R: 5'-GATATTGGATCGGAGTTGCTGAA-3' |
| TOLLIP | F: 5'-TGGGCCGACTGAACATCAC-3' |
|  | R: 5'-GTGGATGACCTTATTCCAGCG-3' |
| DNM1L | F: 5'-CTGCCTCAAATCGTCGTAGTG-3' |
|  | R: 5'-GAGGTCTCCGGGTGACAATTC-3' |
| SNX9 | F: 5'-ACCAAGGCTCGGGTTATGTAT-3' |
|  | R 5'-CCCTCGTTCTCCTTTGATGTTT-3' |
| OPA1 | F: 5'-TGTGAGGTCTGCCAGTCTTTA-3' |
|  | R: 5'-TGTCCTTAATTGGGGTCGTTG-3' |
| α-KGDH | F: 5'-ACATGAGGCCAGATAGGGGA-3' |
|  | R: 5'-TTGGATTACACCGGCCTCAG-3' |
| IDH | F: 5'-GGAGAAGCCGGTAGTGGAGAT-3' |
|  | R: 5'-GGTCTGGTCACGGTTTGGAA-3' |
| CS | F: 5'-AAGGTGGTGGCGTTATACTGC-3' |
|  | R: 5'-CTGGCACAGCGGATGTGAG-3' |
| NDUFB-3 | F: 5'-ACAGACAGTGGAAAATTGAAGGG-3' |
|  | R: 5'-GCCCATGTATCTCCAAGCCT-3' |
| MTCO3 | F: 5'-CCCCAATTAGGAGGGCACTG-3' |
|  | R: 5'-ATGCCGTCGGAAATGGTGAA-3' |
| SDHB | F: 5'-ATTTACCGATGGGACCCAGAC-3' |
|  | R: 5'-GTCCGCACTTATTCAGATCCAC-3' |
| GAPDH | F: 5'-GTCTCCTCTGACTTCAACAGCG-3' |
|  | R: 5'-ACCACCCTGTTGCTGTAGCCAA-3' |

**Table S4**: Reagent Kits

| Reagent Kit Name | Company | Catalogue Number |
| --- | --- | --- |
| BCA protein quantitation assay kit | Beyotime | P0012 |
| RNAiso Plus kit | Takara | 9108Q |
| SYBR Green Real-Time PCR Master Mix | Thermo Fisher | 4309155 |
| TUNEL fluorescence-detection kit | Beyotime | C1086 |
| ROS testing kit | Beyotime | S0033S |
| Cellular lipid peroxidation MDA assay kit | Nanjing Jiancheng Bioengineering Institute | A003-1-2 |
| SOD activity assay kits | Solarbio | BC0170 |
| CAT activity assay kits | Solarbio | BC0200 |
| MitoTracker Red CMXRos | Solarbio | M9940 |
| ATP assay kit | Beyotime | S0026 |
| Lactic acid concentrations with lactate assay kit | Dojindo | L256 |
| Pyruvic acid colorimetric assay kit | Elabscience | E-BC-K130-M |
| PFK-1 activity assay kit | Saint-bio | BA1194 |
| HK activity assay kit | Saint-bio | BA2147 |
| PK activity assay kit | Saint-bio | BA1061 |
| LDH activity assay kit | Saint-bio | BA1260 |
| PDH activity assay kit | Saint-bio | BA1059 |
| Hematoxylin and Eosin Staining Kit | Beyotime | C0105S |
| Masson’s Trichrome Staining Kit | Solarbio | G1340 |
